# Biased-corrected richness estimates for the Amazonian tree flora

**DOI:** 10.1038/s41598-020-66686-3

**Published:** 2020-06-23

**Authors:** Hans ter Steege, Paulo I. Prado, Renato A. F. de Lima, Edwin Pos, Luiz de Souza Coelho, Diogenes de Andrade Lima Filho, Rafael P. Salomão, Iêda Leão Amaral, Francisca Dionízia de Almeida Matos, Carolina V. Castilho, Oliver L. Phillips, Juan Ernesto Guevara, Marcelo de Jesus Veiga Carim, Dairon Cárdenas López, William E. Magnusson, Florian Wittmann, Maria Pires Martins, Daniel Sabatier, Mariana Victória Irume, José Renan da Silva Guimarães, Jean-François Molino, Olaf S. Bánki, Maria Teresa Fernandez Piedade, Nigel C. A. Pitman, José Ferreira Ramos, Abel Monteagudo Mendoza, Eduardo Martins Venticinque, Bruno Garcia Luize, Percy Núñez Vargas, Thiago Sanna Freire Silva, Evlyn Márcia Moraes de Leão Novo, Neidiane Farias Costa Reis, John Terborgh, Angelo Gilberto Manzatto, Katia Regina Casula, Euridice N. Honorio Coronado, Juan Carlos Montero, Alvaro Duque, Flávia R. C. Costa, Nicolás Castaño Arboleda, Jochen Schöngart, Charles Eugene Zartman, Timothy J. Killeen, Beatriz S. Marimon, Ben Hur Marimon-Junior, Rodolfo Vasquez, Bonifacio Mostacedo, Layon O. Demarchi, Ted R. Feldpausch, Julien Engel, Pascal Petronelli, Chris Baraloto, Rafael L. Assis, Hernán Castellanos, Marcelo Fragomeni Simon, Marcelo Brilhante de Medeiros, Adriano Quaresma, Susan G. W. Laurance, Lorena M. Rincón, Ana Andrade, Thaiane R. Sousa, José Luís Camargo, Juliana Schietti, William F. Laurance, Helder Lima de Queiroz, Henrique Eduardo Mendonça Nascimento, Maria Aparecida Lopes, Emanuelle de Sousa Farias, José Leonardo Lima Magalhães, Roel Brienen, Gerardo A. Aymard C., Juan David Cardenas Revilla, Ima Célia Guimarães Vieira, Bruno Barçante Ladvocat Cintra, Pablo R. Stevenson, Yuri Oliveira Feitosa, Joost F. Duivenvoorden, Hugo F. Mogollón, Alejandro Araujo-Murakami, Leandro Valle Ferreira, José Rafael Lozada, James A. Comiskey, José Julio de Toledo, Gabriel Damasco, Nállarett Dávila, Aline Lopes, Roosevelt García-Villacorta, Freddie Draper, Alberto Vicentini, Fernando Cornejo Valverde, Jon Lloyd, Vitor H. F. Gomes, David Neill, Alfonso Alonso, Francisco Dallmeier, Fernanda Coelho de Souza, Rogerio Gribel, Luzmila Arroyo, Fernanda Antunes Carvalho, Daniel Praia Portela de Aguiar, Dário Dantas do Amaral, Marcelo Petratti Pansonato, Kenneth J. Feeley, Erika Berenguer, Paul V. A. Fine, Marcelino Carneiro Guedes, Jos Barlow, Joice Ferreira, Boris Villa, Maria Cristina Peñuela Mora, Eliana M. Jimenez, Juan Carlos Licona, Carlos Cerón, Raquel Thomas, Paul Maas, Marcos Silveira, Terry W. Henkel, Juliana Stropp, Marcos Ríos Paredes, Kyle G. Dexter, Doug Daly, Tim R. Baker, Isau Huamantupa-Chuquimaco, William Milliken, Toby Pennington, J. Sebastián Tello, José Luis Marcelo Pena, Carlos A. Peres, Bente Klitgaard, Alfredo Fuentes, Miles R. Silman, Anthony Di Fiore, Patricio von Hildebrand, Jerome Chave, Tinde R. van Andel, Renato Richard Hilário, Juan Fernando Phillips, Gonzalo Rivas-Torres, Janaína Costa Noronha, Adriana Prieto, Therany Gonzales, Rainiellene de Sá Carpanedo, George Pepe Gallardo Gonzales, Ricardo Zárate Gómez, Domingos de Jesus Rodrigues, Egleé L. Zent, Ademir R. Ruschel, Vincent Antoine Vos, Émile Fonty, André Braga Junqueira, Hilda Paulette Dávila Doza, Bruce Hoffman, Stanford Zent, Edelcilio Marques Barbosa, Yadvinder Malhi, Luiz Carlos de Matos Bonates, Ires Paula de Andrade Miranda, Natalino Silva, Flávia Rodrigues Barbosa, César I. A. Vela, Linder Felipe Mozombite Pinto, Agustín Rudas, Bianca Weiss Albuquerque, Maria Natalia Umaña, Yrma Andreina Carrero Márquez, Geertje van der Heijden, Kenneth R. Young, Milton Tirado, Diego F. Correa, Rodrigo Sierra, Janaina Barbosa Pedrosa Costa, Maira Rocha, Emilio Vilanova Torre, Ophelia Wang, Alexandre A. Oliveira, Michelle Kalamandeen, Corine Vriesendorp, Hirma Ramirez-Angulo, Milena Holmgren, Marcelo Trindade Nascimento, David Galbraith, Bernardo Monteiro Flores, Veridiana Vizoni Scudeller, Angela Cano, Manuel Augusto Ahuite Reategui, Italo Mesones, Cláudia Baider, Casimiro Mendoza, Roderick Zagt, Ligia Estela Urrego Giraldo, Cid Ferreira, Daniel Villarroel, Reynaldo Linares-Palomino, William Farfan-Rios, William Farfan-Rios, Luisa Fernanda Casas, Sasha Cárdenas, Henrik Balslev, Armando Torres-Lezama, Miguel N. Alexiades, Karina Garcia-Cabrera, Luis Valenzuela Gamarra, Elvis H. Valderrama Sandoval, Freddy Ramirez Arevalo, Lionel Hernandez, Adeilza Felipe Sampaio, Susamar Pansini, Walter Palacios Cuenca, Edmar Almeida de Oliveira, Daniela Pauletto, Aurora Levesley, Karina Melgaço, Georgia Pickavance

**Affiliations:** 1 Naturalis Biodiversity Center, PO Box 9517, Leiden, 2300 RA The Netherlands; 2 Systems Ecology, Vrije Universiteit Amsterdam, De Boelelaan 1087, Amsterdam, 1081 HV The Netherlands; 3 Instituto de Biociências - Dept. Ecologia, Universidade de Sao Paulo - USP, Rua do Matão, Trav. 14, no. 321, Cidade Universitária, São Paulo, SP 05508-090 Brazil; 4 Ecology & Biodiversity Group, Utrecht University, Padualaan 8, Utrecht, 3584 CH The Netherlands; 5 Coordenação de Biodiversidade, Instituto Nacional de Pesquisas da Amazônia - INPA, Av. André Araújo, 2936, Petrópolis, Manaus, AM 69067-375 Brazil; 6 Programa Professor Visitante Nacional Sênior na Amazônia - CAPES, Universidade Federal Rural da Amazônia, Av. Perimetral, s/n, Belém, PA Brazil; 7 Coordenação de Botânica, Museu Paraense Emílio Goeldi, Av. Magalhães Barata 376, C.P. 399, Belém, PA 66040-170 Brazil; 8 EMBRAPA – Centro de Pesquisa Agroflorestal de Roraima, BR 174, km 8 – Distrito Industrial, Boa Vista, RR 69301-970 Brazil; 9 School of Geography, University of Leeds, Woodhouse Lane, Leeds, LS2 9JT UK; 10 Grupo de Investigación en Biodiversidad, Medio Ambiente y Salud-BIOMAS, Universidad de las Américas, Campus Queri, Quito, Ecuador; 11 Keller Science Action Center, The Field Museum, 1400 S. Lake Shore Drive, Chicago, IL 60605-2496 USA; 12 Departamento de Botânica, Instituto de Pesquisas Científicas e Tecnológicas do Amapá - IEPA, Rodovia JK, Km 10, Campus do IEPA da Fazendinha, Amapá, 68901-025 Brazil; 13 Herbario Amazónico Colombiano, Instituto SINCHI, Calle 20 No 5-44, Bogotá, DC Colombia; 14 Coordenação de Pesquisas em Ecologia, Instituto Nacional de Pesquisas da Amazônia - INPA, Av. André Araújo, 2936, Petrópolis, Manaus, AM 69067-375 Brazil; 15 Dep. of Wetland Ecology, Institute of Geography and Geoecology, Karlsruhe Institute of Technology - KIT, Josefstr.1, Rastatt, D-76437 Germany; 16 Biogeochemistry, Max Planck Institute for Chemistry, Hahn-Meitner Weg 1, Mainz, 55128 Germany; 17 AMAP, IRD, Cirad, CNRS, INRA, Université de Montpellier, Montpellier, F-34398 France; 18 Coordenação de Dinâmica Ambiental, Instituto Nacional de Pesquisas da Amazônia - INPA, Av. André Araújo, 2936, Petrópolis, Manaus, AM 69067-375 Brazil; 19 Science and Education, The Field Museum, 1400 S. Lake Shore Drive, Chicago, IL 60605-2496 USA; 20 Jardín Botánico de Missouri, Oxapampa, Pasco, Peru; 21 Centro de Biociências, Departamento de Ecologia, Universidade Federal do Rio Grande do Norte, Av. Senador Salgado Filho, 3000, Natal, RN 59072-970 Brazil; 22 Departamento de Ecologia, Universidade Estadual Paulista - UNESP – Instituto de Biociências – IB, Av. 24 A, 1515, Bela Vista, Rio Claro, SP 13506-900 Brazil; 23 Herbario Vargas, Universidad Nacional de San Antonio Abad del Cusco, Avenida de la Cultura, Nro 733, Cusco, Cuzco Peru; 24 Biological and Environmental Sciences, University of Stirling, Stirling, FK9 4LA UK; 25 Divisao de Sensoriamento Remoto – DSR, Instituto Nacional de Pesquisas Espaciais – INPE, Av. dos Astronautas, 1758, Jardim da Granja, São José dos Campos, SP 12227-010 Brazil; 26 Programa de Pós- Graduação em Biodiversidade e Biotecnologia PPG- Bionorte, Universidade Federal de Rondônia, Campus Porto Velho Km 9,5 bairro Rural, Porto Velho, RO 76.824-027 Brazil; 27 Department of Biology and Florida Museum of Natural History, University of Florida, Gainesville, FL 32611 USA; 28 Centre for Tropical Environmental and Sustainability Science and College of Science and Engineering, James Cook University, Cairns, Queensland 4870 Australia; 29 Departamento de Biologia, Universidade Federal de Rondônia, Rodovia BR 364 s/n Km 9,5 - Sentido Acre, Unir, Porto Velho, RO 76.824-027 Brazil; 30 Instituto de Investigaciones de la Amazonía Peruana (IIAP), Av. A. Quiñones km 2,5, Iquitos, Loreto 784 Peru; 31 Instituto Boliviano de Investigacion Forestal, Av. 6 de agosto #28, Km. 14, Doble via La Guardia, Casilla 6204, Santa Cruz, Santa Cruz Bolivia; 32 Departamento de Ciencias Forestales, Universidad Nacional de Colombia, Calle 64 x Cra 65, Medellín, Antioquia 1027 Colombia; 33 Agteca-Amazonica, Santa Cruz, Bolivia; 34 Programa de Pós-Graduação em Ecologia e Conservação, Universidade do Estado de Mato Grosso, Nova Xavantina, MT Brazil; 35 Facultad de Ciencias Agrícolas, Universidad Autónoma Gabriel René Moreno, Santa Cruz, Santa Cruz Bolivia; 36 Geography, College of Life and Environmental Sciences, University of Exeter, Rennes Drive, Exeter EX4 4RJ UK; 37 International Center for Tropical Botany (ICTB) Department of Biological Sciences, Florida International University, 11200 SW 8th Street, OE 243, Miami, FL 33199 USA; 38 Cirad UMR Ecofog, AgrosParisTech,CNRS,INRA,Univ Guyane, Campus agronomique, Kourou Cedex, 97379 France; 39 Natural History Museum, University of Oslo, Postboks 1172, Oslo, 0318 Norway; 40 Centro de Investigaciones Ecológicas de Guayana, Universidad Nacional Experimental de Guayana, Calle Chile, urbaniz Chilemex, Puerto Ordaz, Bolivar, Venezuela; 41 Prédio da Botânica e Ecologia, Embrapa Recursos Genéticos e Biotecnologia, Parque Estação Biológica, Av. W5 Norte, Brasilia, DF 70770-917 Brazil; 42 Projeto Dinâmica Biológica de Fragmentos Florestais, Instituto Nacional de Pesquisas da Amazônia - INPA, Av. André Araújo, 2936, Petrópolis, Manaus, AM 69067-375 Brazil; 43 Diretoria Técnico-Científica, Instituto de Desenvolvimento Sustentável Mamirauá, Estrada do Bexiga, 2584, Tefé, AM 69470-000 Brazil; 44 Instituto de Ciências Biológicas, Universidade Federal do Pará, Av. Augusto Corrêa 01, Belém, PA 66075-110 Brazil; 45 Laboratório de Ecologia de Doenças Transmissíveis da Amazônia (EDTA), Instituto Leônidas e Maria Deane, Fiocruz, Rua Terezina, 476, Adrianópolis, Manaus, AM 69060-001 Brazil; 46 Programa de Pós-graduação em Biodiversidade e Saúde, Instituto Oswaldo Cruz - IOC/FIOCRUZ, Pav. Arthur Neiva – Térreo, Av. Brasil, 4365 – Manguinhos, Rio de Janeiro, RJ 21040-360 Brazil; 47 Programa de Pós-Graduação em Ecologia, Universidade Federal do Pará, Av. Augusto Corrêa 01, Belém, PA 66075-110 Brazil; 48 Embrapa Amazônia Oriental, Trav. Dr. Enéas Pinheiro s/n°, Belém, PA 66095-100 Brazil; 49 Programa de Ciencias del Agro y el Mar, Herbario Universitario (PORT), UNELLEZ-Guanare, Guanare, Portuguesa 3350 Venezuela; 50 Instituto de Biociências - Dept. Botanica, Universidade de Sao Paulo - USP, Rua do Matão 277, Cidade Universitária, São Paulo, SP 05508-090 Brazil; 51 Laboratorio de Ecología de Bosques Tropicales y Primatología, Universidad de los Andes, Carrera 1 # 18a- 10, Bogotá, DC 111711 Colombia; 52 Programa de Pós-Graduação em Biologia (Botânica), Instituto Nacional de Pesquisas da Amazônia - INPA, Av. André Araújo, 2936, Petrópolis, Manaus, AM 69067-375 Brazil; 53 Institute of Biodiversity and Ecosystem Dynamics, University of Amsterdam, Sciencepark 904, Amsterdam, 1098 XH The Netherlands; 54 Endangered Species Coalition, 8530 Geren Rd., Silver Spring, MD 20901 USA; 55 Museo de Historia Natural Noel Kempff Mercado, Universidad Autónoma Gabriel Rene Moreno, Avenida Irala 565 Casilla Post al 2489, Santa Cruz, Santa Cruz Bolivia; 56 Facultad de Ciencias Forestales y Ambientales, Instituto de Investigaciones para el Desarrollo Forestal, Universidad de los Andes, Via Chorros de Milla, 5101 Mérida, Mérida Venezuela; 57 Inventory and Monitoring Program, National Park Service, 120 Chatham Lane, Fredericksburg, VA 22405 USA; 58 Center for Conservation and Sustainability, Smithsonian Conservation Biology Institute, 1100 Jefferson Dr. SW, Suite 3123, Washington, DC 20560-0705 USA; 59 Universidade Federal do Amapá, Ciências Ambientais, Rod. Juscelino Kubitschek km2, Macapá, AP 68902-280 Brazil; 60 Department of Integrative Biology, University of California, Berkeley, CA 94720-3140 USA; 61 Biologia Vegetal, Universidade Estadual de Campinas, Caixa Postal 6109, Campinas, SP 13.083-970 Brazil; 62 Department of Ecology, University of Brasilia, Brasilia, DF 70904-970 Brazil; 63 Department of Ecology and Evolutionary Biology, Cornell University, Corson Hall, 215 Tower Road, Ithaca, NY 14850 USA; 64 Peruvian Center for Biodiversity and Conservation (PCBC), Iquitos, Peru; 65 Department of Global Ecology, Carnegie Institution for Science, 260 Panama St., Stanford, CA 94305 USA; 66 Andes to Amazon Biodiversity Program, Madre de Dios, Madre de Dios Peru; 67 Faculty of Natural Sciences, Department of Life Sciences, Imperial College London, Silwood Park, South Kensington Campus, London, SW7 2AZ UK; 68 Escola de Negócios Tecnologia e Inovação, Centro Universitário do Pará, Belém, PA Brazil; 69 Universidade Federal do Pará, Rua Augusto Corrêa 01, Belém, PA 66075-110 Brazil; 70 Ecosistemas, Biodiversidad y Conservación de Especies, Universidad Estatal Amazónica, Km. 2 1/2 vía a Tena (Paso Lateral), Puyo, Pastaza Ecuador; 71 Universidade Federal de Minas Gerais, Instituto de Ciências Biológicas, Departamento de Genética, Ecologia e Evolução, Av. Antônio Carlos, 6627 Pampulha, Belo Horizonte, MG 31270-901 Brazil; 72 Department of Biology, University of Miami, Coral Gables, FL 33146 USA; 73 Fairchild Tropical Botanic Garden, Coral Gables, FL 33156 USA; 74 Environmental Change Institute, University of Oxford, Oxford, Oxfordshire OX1 3QY UK; 75 Lancaster Environment Centre, Lancaster University, Lancaster, Lancashire LA1 4YQ UK; 76 Empresa Brasileira de Pesquisa Agropecuária, Embrapa Amapá, Rod. Juscelino Kubitschek km 5, Macapá, Amapá 68903-419 Brazil; 77 Direccíon de Evaluación Forestal y de Fauna Silvestre, Av. Javier Praod Oeste 693, Magdalena del Mar, Peru; 78 Universidad Regional Amazónica IKIAM, Km 7 via Muyuna, Tena, Napo Ecuador; 79 Grupo de Ecología y Conservación de Fauna y Flora Silvestre, Instituto Amazónico de Investigaciones Imani, Universidad Nacional de Colombia sede Amazonia, Leticia, Amazonas Colombia; 80 Escuela de Biología Herbario Alfredo Paredes, Universidad Central, Ap. Postal 17.01.2177, Quito, Pichincha Ecuador; 81 Iwokrama International Centre for Rain Forest Conservation and Development, Georgetown, Guyana; 82 Museu Universitário/Centro de Ciências Biológicas e da Natureza/Laboratório de Botânica e Ecologia Vegetal, Universidade Federal do Acre, Rio Branco, AC 69915-559 Brazil; 83 Department of Biological Sciences, Humboldt State University, 1 Harpst Street, Arcata, CA 95521 USA; 84 Institute of Biological and Health Sciences, Federal University of Alagoas, Av. Lourival Melo Mota, s/n, Tabuleiro do Martins, Maceio, AL 57072-970 Brazil; 85 Servicios de Biodiversidad EIRL, Jr. Independencia 405, Iquitos, Loreto 784 Peru; 86 School of Geosciences, University of Edinburgh, 201 Crew Building, King’s Buildings, Edinburgh, EH9 3JN UK; 87 Tropical Diversity Section, Royal Botanic Garden Edinburgh, 20a Inverleith Row, Edinburgh, Scotland EH3 5LR UK; 88 New York Botanical Garden, 2900 Southern Blvd, Bronx, New York, NY 10458-5126 USA; 89 Natural Capital and Plant Health, Royal Botanic Gardens, Kew, Richmond, Surrey TW9 3AB UK; 90 Center for Conservation and Sustainable Development, Missouri Botanical Garden, P.O. Box 299, St. Louis, MO 63166-0299 USA; 91 Universidad Nacional de Jaén, Carretera Jaén San Ignacio Km 23, Jaén, Cajamarca 06801 Peru; 92 School of Environmental Sciences, University of East Anglia, Norwich, NR4 7TJ UK; 93 Department for Identification & Naming, Royal Botanic Gardens, Kew, Richmond, Surrey, TW9 3AB UK; 94 Herbario Nacional de Bolivia, Universitario UMSA, Casilla 10077 Correo Central, La Paz, La Paz Bolivia; 95 Biology Department and Center for Energy, Environment and Sustainability, Wake Forest University, 1834 Wake Forest Rd, Winston Salem, NC 27106 USA; 96 Department of Anthropology, University of Texas at Austin, SAC 5.150, 2201 Speedway Stop C3200, Austin, TX 78712 USA; 97 Fundación Estación de Biología, Cra 10 No. 24-76 Oficina 1201, Bogotá, DC Colombia; 98 Laboratoire Evolution et Diversité Biologique, CNRS and Université Paul Sabatier, UMR 5174 EDB, Toulouse, 31000 France; 99 Biosystematics group, Wageningen University, Droevendaalsesteeg 1, Wageningen, 6708 PB The Netherlands; 100 Fundación Puerto Rastrojo, Cra 10 No. 24-76 Oficina 1201, Bogotá, DC Colombia; 101 Colegio de Ciencias Biológicas y Ambientales-COCIBA & Galapagos Institute for the Arts and Sciences-GAIAS, Universidad San Francisco de Quito-USFQ, Quito, Pichincha Ecuador; 102 Department of Wildlife Ecology and Conservation, University of Florida, 110 Newins-Ziegler Hall, Gainesville, FL 32611 USA; 103 ICNHS, Federal University of Mato Grosso, Av. Alexandre Ferronato 1200, Setor Industrial, Sinop, MT 78.557-267 Brazil; 104 Instituto de Ciencias Naturales, Universidad Nacional de Colombia, Apartado 7945, Bogotá, DC Colombia; 105 ACEER Foundation, Jirón Cusco N° 370, Puerto Maldonado, Madre de Dios Peru; 106 PROTERRA, Instituto de Investigaciones de la Amazonía Peruana (IIAP), Av. A. Quiñones km 2,5, Iquitos, Loreto 784 Peru; 107 Laboratory of Human Ecology, Instituto Venezolano de Investigaciones Científicas - IVIC, Ado 20632, Caracas, DC 1020 A Venezuela; 108 Universidad Autónoma del Beni José Ballivián, Campus Universitario Final, Av. Ejercito, Riberalta, Beni Bolivia; 109 Direction régionale de la Guyane, ONF, Cayenne, F-97300 French Guiana; 110 Institut de Ciència i Tecnologia Ambientals, Universitat Autònoma de Barcelona, 08193 Bellaterra Barcelona, Spain; 111 Amazon Conservation Team, Doekhieweg Oost #24, Paramaribo, Suriname; 112 Environmental Change Institute, Oxford University Centre for the Environment, Dyson Perrins Building, South Parks Road, Oxford, England OX1 3QY UK; 113 Instituto de Ciência Agrárias, Universidade Federal Rural da Amazônia, Av. Presidente Tancredo Neves 2501, Belém, PA 66.077-830 Brazil; 114 Escuela Profesional de Ingeniería Forestal, Universidad Nacional de San Antonio Abad del Cusco, Jirón San Martín 451, Puerto Maldonado, Madre de Dios Peru; 115 Department of Ecology and Evolutionary Biology, University of Michigan, Ann Arbor, MI 48109 USA; 116 University of Nottingham, University Park, Nottingham NG7 2RD UK; 117 Geography and the Environment, University of Texas at Austin, 305 E. 23rd Street, CLA building, Austin, TX 78712 USA; 118 GeoIS, El Día 369 y El Telégrafo, 3° Piso, Quito, Pichincha Ecuador; 119 School of Agriculture and Food Sciences - ARC Centre of Excellence for Environmental Decisions CEED, The University of Queensland, St. Lucia, QLD 4072 Australia; 120 Instituto de Investigaciones para el Desarrollo Forestal (INDEFOR), Universidad de los Andes, Conjunto Forestal, 5101 Mérida, Mérida Venezuela; 121 School of Environmental and Forest Sciences, University of Washington, Seattle, WA USA; 122 Environmental Science and Policy, Northern Arizona University, Flagstaff, AZ 86011 USA; 123 Laurentian University, 935 Ramsey Lake Road, Sudbury, Ontario P3E 2C6 Canada; 124 Resource Ecology Group, Wageningen University & Research, Droevendaalsesteeg 3a, Lumen, building number 100, Wageningen, Gelderland 6708 PB The Netherlands; 125 Laboratório de Ciências Ambientais, Universidade Estadual do Norte Fluminense, Av. Alberto Lamego 2000, Campos dos Goyatacazes, RJ 28013-620 Brazil; 126 University of Campinas, Plant Biology Department, Rua Monteiro Lobato, 255, Cidade Universitária Zeferino Vaz, Barão Geraldo, Campinas, São Paulo, CEP 13083-862 Brazil; 127 Departamento de Biologia, Universidade Federal do Amazonas - UFAM – Instituto de Ciências Biológicas – ICB1, Av General Rodrigo Octavio 6200, Manaus, AM 69080-900 Brazil; 128 Cambridge University Botanic Garden, 1 Brookside, Cambridge, CB2 1JE UK; 129 Medio Ambiente, PLUSPRETOL, Iquitos, Loreto Peru; 130 The Mauritius Herbarium, Agricultural Services, Ministry of Agro-Industry and Food Security, Reduit, 80835 Mauritius; 131 Escuela de Ciencias Forestales (ESFOR), Universidad Mayor de San Simon (UMSS), Sacta, Cochabamba Bolivia; 132 FOMABO, Manejo Forestal en las Tierras Tropicales de Bolivia, Sacta, Cochabamba Bolivia; 133 Tropenbos International, Lawickse Allee 11 PO Box 232, Wageningen, 6700 AE The Netherlands; 134 Living Earth Collaborative, Washington University in Saint Louis, St. Louis, MO 63130 USA; 135 Department of Bioscience, Aarhus University, Building 1540 Ny Munkegade, Aarhus C, Aarhus, DK 8000 Denmark; 136 School of Anthropology and Conservation, University of Kent, Marlowe Building, Canterbury, Kent, CT2 7NR UK; 137 Department of Biology, University of Missouri, St. Louis, MO 63121 USA; 138 Facultad de Biologia, Universidad Nacional de la Amazonia Peruana, Pevas 5ta cdra, Iquitos, Loreto Peru; 139 Herbario Nacional del Ecuador, Universidad Técnica del Norte, Quito, Pichincha Ecuador; 140 Instituto de Biodiversidade e Floresta, Universidade Federal do Oeste do Pará, Rua Vera Paz, Campus Tapajós, Santarém, PA 68015-110 Brazil

**Keywords:** Community ecology, Theoretical ecology

## Abstract

Amazonian forests are extraordinarily diverse, but the estimated species richness is very much debated. Here, we apply an ensemble of parametric estimators and a novel technique that includes conspecific spatial aggregation to an extended database of forest plots with up-to-date taxonomy. We show that the species abundance distribution of Amazonia is best approximated by a logseries with aggregated individuals, where aggregation increases with rarity. By averaging several methods to estimate total richness, we confirm that over 15,000 tree species are expected to occur in Amazonia. We also show that using ten times the number of plots would result in an increase to just ~50% of those 15,000 estimated species. To get a more complete sample of all tree species, rigorous field campaigns may be needed but the number of trees in Amazonia will remain an estimate for years to come.

## Introduction

The lowland rainforest of the Amazon River basin and Guiana Shield, hereafter Amazonia, covers an area of nearly 6 million km^2^ with an estimated total number of 3.9 × 10^11^ trees (diameter at 1.30 m - dbh ≥ 10 cm)^1^. Sampling such an extensive area has been extremely limited, and accurate estimates of the total number of tree species and their populations have thus been difficult to obtain^1^. Nevertheless, these estimates are important to understand the difference between what we already know and what still needs to be discovered about Amazonian tree diversity. In 2013, we estimated that around 16,000 tree species should occur within Amazonia^1^ using the distribution of estimated total abundances of all tree species occurring in 1,170 forest plots scattered across the area. A number that has both been criticized^2–4^ and accepted as plausible for plot inventories^5^. In the following years there has been considerable progress both in the taxonomy of Amazonian tree species^4,6–8^ and the number of forest inventory plots available^9^, which has steadily grown from 1,170 to 1,946. In addition, our previous richness estimate was based on a single estimation method, the linear extension of a hypothetical logseries^10^ of the estimated population sizes of the Amazonian tree species present in our plots^1^.

While the logseries fits the relative abundance distribution of many taxa quite well^11^, others have recently argued that it tends to overestimate richness and that other models such as the negative binomial would provide better estimates^12^. Other parametric methods, such as the Poisson lognormal^13,14^ have also been suggested to have a better performance than the logseries when used to describe species relative abundances in Amazonian forest^15^.

Here we present a new species richness estimation of Amazonian tree species based on the revised taxonomy and increased inventory dataset using various parametric estimation methods. Non-parametric methods^4,16^ continue to be used to estimate species richness but as they tend to hugely underestimate richness in sparsely sampled, species-rich areas^6,17,18^, such as Amazonia, we did not employ them here. All methods assume random sampling of species with random distribution across space, conditions mostly not met in forest inventory data. Rather, limited dispersal and ecological preferences of trees tend to result in aggregated spatial patterns of species distribution^19–21^. Hence, conspecific aggregation at the sampling scale has also been pointed out as a source of serious bias of parametric estimates of species richness^12,21^.

The new advances in species taxonomy, improved sampling coverage and richness estimation methods, discussed above, allow us to provide an update on the estimated number of species in the Amazonian tree flora and their estimated population sizes, which are key to the understanding and conservation of the Amazonian tree diversity. Making use of the March 2019 Amazon Tree Diversity Network (ATDN) database, an improved Amazon lowland forest map^22^, and an updated taxonomy^4,8^, we provide new estimates of the (i) population sizes for each species and (ii) total species richness of the Amazonian tree flora. We provide species richness estimates using parametric methods based on the logseries (LS), negative binomial (NB) and Poisson lognormal (PLN). For the first time, we use data on species occurrences across plots to assess the impacts of conspecific aggregation in the estimation of tree species richness in Amazonia. Based on the simulation of the sampling from a hypothetical species abundance distribution (SAD) for the Amazonian tree flora, we evaluate the accuracy of each estimation method and provide correction of biases in their estimates. Finally, we evaluate how robust the richness estimates of each method are to increases in the number of forest plots included in the sample and to changes in species taxonomy.

## Results

### Raw data description

The 2019 version of the dataset (1,946 plots) contained a total of 1,101,368 individuals, 89% of which were identified to a valid species name (Supplementary Table 1). It contained a total of 5,027 tree species (Appendix 1), representing an increase of 63 observed species in comparison to the 2013 version (496 species, if the 2013 dataset would have used the current taxonomy – see also Appendix 2, 3). The total number of trees in Amazonia, based on the tree density modelling, was estimated at 3.06 ∙ 10^11^. The 10 most common species were: *Eschweilera coriacea*, *Euterpe precatoria*, *Oenocarpus bataua*, *Pseudolmedia laevis*, *Protium altissimum*, *Iriartea deltoidea*, *Mauritia flexuosa*, *Socratea exorrhiza*, *Astrocaryum murumuru*, and *Pentaclethra macroloba* (species authorities follow ref. ^8^). Six of these species are palms (see Appendix 1 for the population estimates for all species). Most of the hyperdominant species show only small changes in their estimated populations compared to 2013 and only small differences in rank.

### Fitted models to empirical SADs

**Figure 1:**
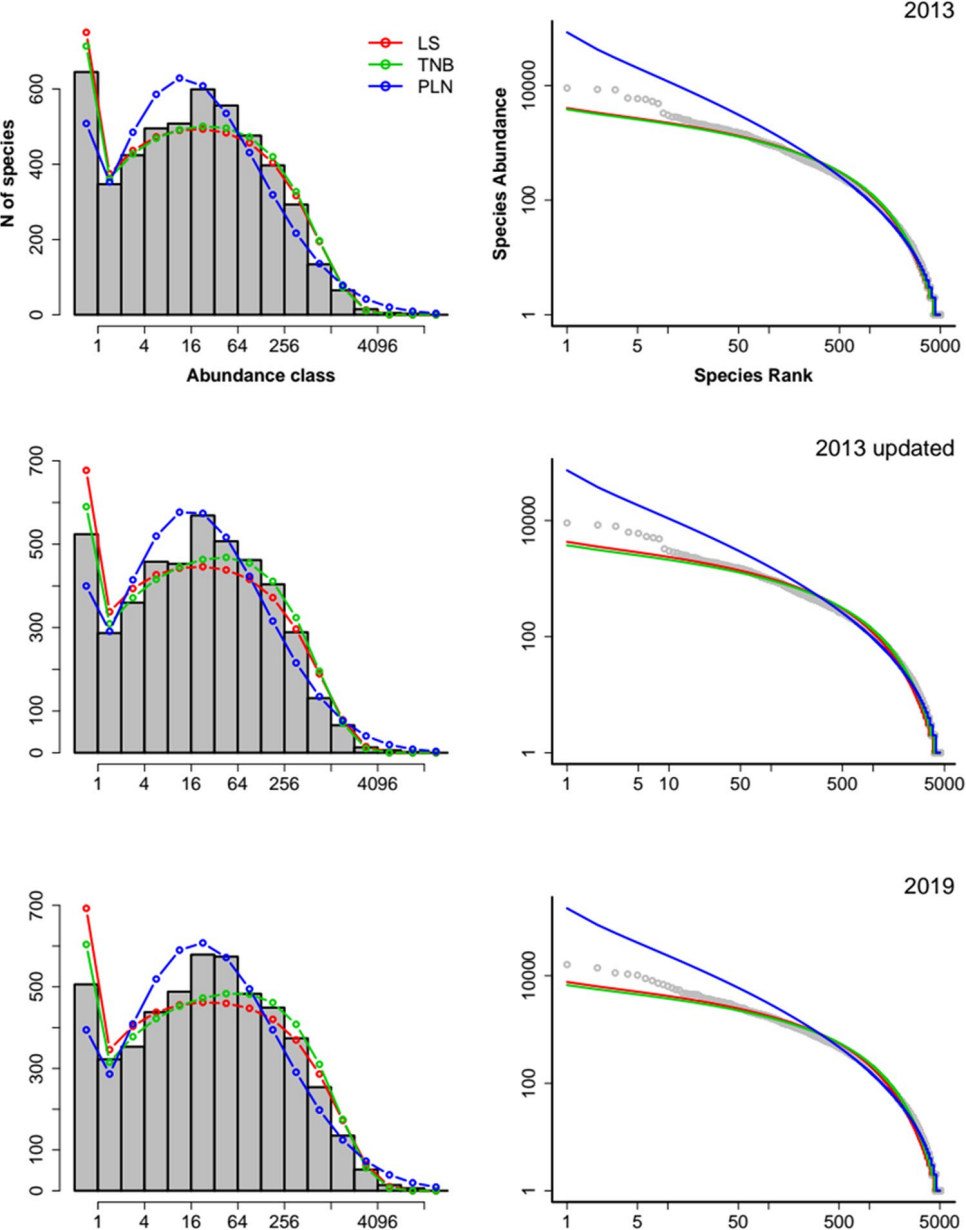
Fit of SAD models to the species abundances from samples from the three ATDN data sets. Left column: observed frequencies of species in each abundance class (octaves, grey bars) and frequencies predicted by analytical Logseries (LS), truncated negative binomial (TNB) and Poisson-lognormal (PLN). Right column: rank-abundance plot in log-log scale of the abundances of species (gray) and the predicted abundances at each rank by the same three models.

The truncated negative binomial (TNB) was the model with best fit to the empirical SADs for all versions of the data sets (Table 1, Supplementary Table 2) with the logseries providing an equally good fit for the 2013 dataset (Table 1, Supplementary Fig. 1), if we used AIC. Model selection with a Bayesian Information Criterion (BIC) had the same result, except by the best support of LS for the 2013 data set (Supplementary Table 3). Visually, the LS and TNB provided very similar fits, with both models underestimating the abundances of the most abundant species in the sample (Fig. 1). The PLN model had no support from the three data sets, overestimating the abundance of common species and underestimating the abundance of rare ones (Fig. 1).

**Table 1:** Delta-AIC for each parametric model fitted to the empirical SAD constructed using the 2013 version of the data (less plots, old taxonomy), the updated 2013 version (less plots, updated taxonomy) and the 2019 version (more plots, updated taxonomy).

Model	DF	2013	2013 updated	2019
Negative Binomial (TNB)	2	0.00	0.00	0.00
Logseries (LS)	1	1.67	30.02	31.52
Poisson-lognormal (PLN)	2	60.43	47.87	42.09

For each dataset version, the better supported model has 0 Delta-AIC. Models with Delta-AIC < 2 have equal support. DF = number of parameters in the model.

### Estimated species richness

The number of species estimated greatly depended on the method of estimation. The original estimates provided by each method, could differ by a factor three from one another (Supplementary Table 4, Supplementary Fig. 2). Because the PLN had little or no support from the data and provided richness estimates (5,649) that were much smaller than species already collected in Amazonia, this method was not further considered. The TNB estimate for the 2013 dataset was similar to those found by ref. ^11^. (13,602 ± 711, compared with 13,497 and range estimates of 14,324 – 12,448, Supplementary Fig. 2, Supplementary Table 4).

**Figure 2:**
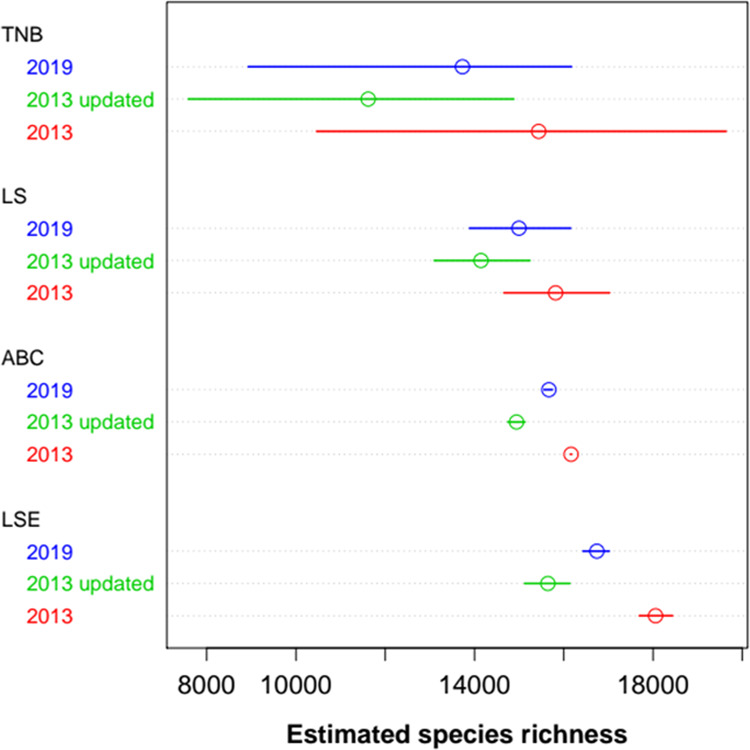
Bias-corrected estimates of total species richness for each method and each data set. TNB, LS: upscaling from the fit of Truncated Negative Binomial and the analytical Logseries to abundances in the sample; LSE: linear extension of the distribution of estimated population sizes; ABC: approximated Bayesian Computation for estimated population sizes distribution. ABC and all bias corrections are derived from simulated samples with conspecific clumping from a logseries community. Bars depict bias-corrected 95% confidence intervals or similar (credible interval for ABC).

**Figure 3:**
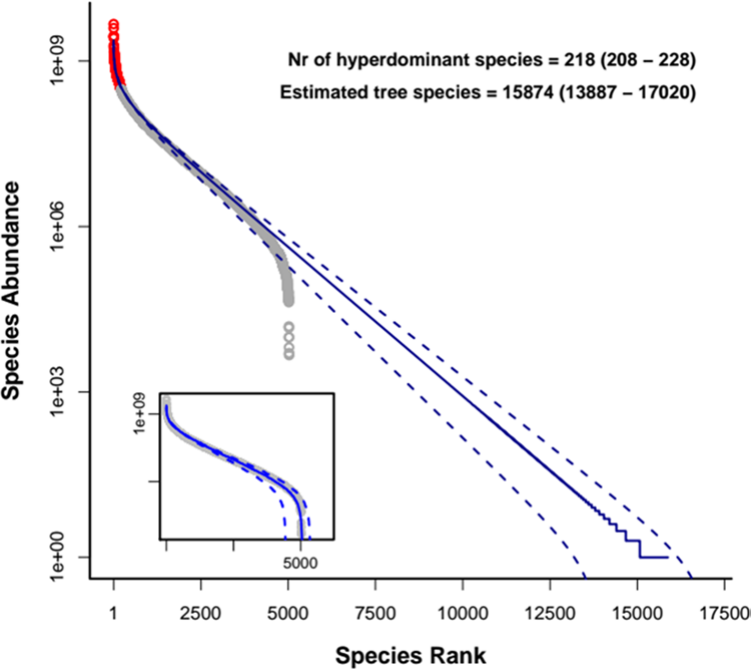
Extension of assumed logseries of estimated population sizes to predict the number of species in Amazonian forests. Grey dots in both panels are the estimated total population sizes of species recorded in the 2019 ATDN data set. The solid blue line in the main figure is the rank-abundance relationship predicted by a logseries with the average of estimates of number of species (15,874 spp). Dotted lines in the main panel delimit the rank-abundance for minimum and maximum of lower and upper limits 95% of the estimates. The lines in the inset panel are the mean and lower and upper bounds for the values of abundances estimates of the recorded species, also from averaging over all estimation methods.

We found that conspecific aggregation introduced bias in the estimation of the species richness for the LS, TNB, and Logseries expansion method (LSE), resulting in an underestimation of the true species richness (Supplementary Table 4, Supplementary Fig. 3). Hence, the original richness estimates had to be corrected to provide more accurate estimates of the richness. The TNB provided the most discrepant and uncertain bias-corrected estimates, while the other methods provided comparable numbers (Fig. 2, Supplementary Fig. 4). This low precision of corrected TNB estimates was caused by a non-linear relationship between estimated and true values (Supplementary Fig. 3a–c). Excluding TNB due to this low precision, the average of estimates for the 2019 dataset, weighted by the inverse of their standard errors, was 15,835 tree species for the Amazonian tree flora. As a conservative interval estimate, we can use the minimum and maximum of the range estimates, also excluding TNB: 13,887 to 17,020 species (Fig. 3). This weighted average estimate for the original 2013 data set was 16,243 (14,659–18,439) species, and 15,020 (13,095–16,136) species for the reviewed 2013 data set.

The LS model had no support in simulated samples drawn from logseries regional SADs with more than 10,000 species with clumping, but had a constant and high support in simulated samples without clumping (Supplementary Fig. 5). For simulated samples from a Negative Binomial regional SAD, the LS model had a poor support for communities with more than 10,000 species, irrespective of conspecific clumping. Thus, the conspecific clumping observed in our data sets causes a strong selection bias against LS, when this is the correct model, but does not cause selection bias for TNB. The best support of TNB provided by the abundances in the sample could thus be an artefact. In fact, the approximate Bayesian Computation model selection showed that LS models had by far the largest posterior probabilities to approximate the distribution of total population sizes, for all three data sets (Table 2).

**Table 2:** ABC model selection. Posterior probabilities of each combination of regional SAD (PLN, LS, TNB) and conspecific distribution (clumped or random) in simulated samples to approximate observed distribution of estimated population sizes, for the three datasets.

Data set	Poisson-lognormal		Logseries		Negative Binomial	
	Clumped	Random	Clumped	Random	Clumped	Random
2013	0.00	0.00	0.71	0.29	0.00	0.00
2013 updated	0.00	0.00	0.76	0.24	0.00	0.00
2019	0.00	0.00	0.91	0.09	0.00	0.00

ABC posterior probabilities estimates the probability of each simulation model to output an acceptable approximation of empirical data. Sizes of posterior samples: 600 (2013), 508(2013 updated) and 570 (2019).

For all datasets, simulations of samples with conspecific clumping of a logseries regional SAD had the highest posterior Approximate Bayesian Computation (ABC) probabilities, and simulations of samples from a truncated negative binomial or a lognormal had very low or zero posterior probabilities (Table 2). We thus assumed the simulations of sampling of a logseries regional SAD with conspecific aggregation as the best approximation of the process that generated the ATDN data.

### Impacts of sampling effort and species taxonomy

Bias-corrected estimates of species richness, using conspecific aggregation fell between 13,730 (TNB) and 16,741 (LSE) species for the 2019 dataset, a variation of 22% (Fig. 2, Supplementary Table 4). Corresponding figures for the 2013 and updated 2013 data sets were 15,437 – 18,056 (TNB - LSE, 20%) and 11,618 – 15,643 (TNB - LSE, 35%) respectively. The taxonomic update of the 2013 data set led to a decrease in the number and abundance of rarer species (species below the median abundance ranking and with densities below to 2–5 individuals/ha in the sample, Supplementary Fig. 6). The same effect was observed in the distribution of abundances of estimated population sizes (Supplementary Fig. 7). As a consequence, the taxonomic update decreased the estimated number of species by all methods. This reduction ranged from –7.6% (ABC) to –24.8% (TNB). The expansion of inventory plots for the 2019 ATDN database increased the number and abundance of rarer species (Supplementary Figs. 6 and 7), which in turn partially reversed the decreasing of the estimated numbers of species (Fig. 2), increasing the estimated richness by 4.9% (ABC) to 18.1% (TNB). The sensitivity of the methods to taxonomic updates and to the expansion of the database followed same order: ABC < LS < LSE < TNB (Fig. 2). Overall, there was more variation of estimates among methods than among the versions of the database. The variance components of the point estimates were 52% for estimation methods and 38% for the data sets, with a residual component of 10%.

### Predicted richness with increasing sample sizes

**Figure 4:**
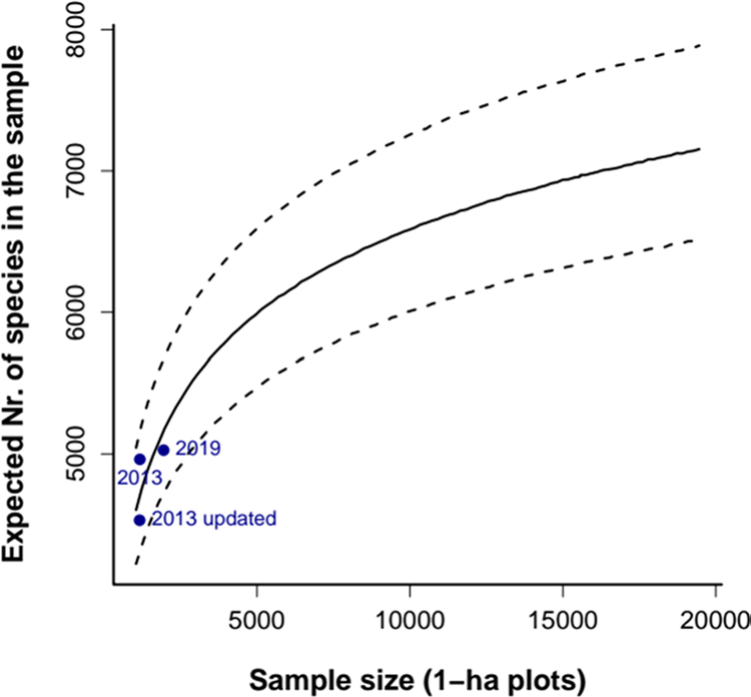
Expected species-accumulation curves from simulated samples logseries with conspecific clumping based on the 2019 data set. The lines show simulated samples of a logseries with the mean (solid line) and lower-upper bounds (dotted lines) of the estimated number of species in the plot sample. Blue dots show the observed values for the three data sets.

We predicted species richness for larger plot samples, using a logseries with 15,874 species, which was the average estimation of total species richness for Amazonia from the 2019 data set. Two other logseries with 13,887 and 17,020 species respectively, were used as the lower and upper bounds for the estimated number of species. The simulations predicted that on average 746 additional species would be recorded in the plots if the current sample size was doubled, an increase of 15% (lower and upper bounds: 247 – 1,317, or 5–26%, Fig. 4). The expected number species for the same sample size, assuming random dispersion of all species, can be estimated with equation S.3. Assuming a mean density of 528.5 trees ha-1 in Amazon that would amount to 6,110 species, or an increase of 22%. As expected, conspecific clumping, which is stronger for the rare species (Supplementary Fig. 8), decreases the rates of accumulation of species.

If the number of plots was ten times the current sample size, the expected number of species in the plots would be 6,958 (an increase of 38%; lower and upper bounds: 6,517 – 7,884), getting close to half of the species estimated to be present in Amazonia.

## Discussion

Our ability to estimate species richness ultimately depends on the capacity to accurately describe patterns of commonness and rarity from samples of local communities and project it to a much larger sample size (see also ref. ^18^). This is not a trivial challenge and it has been the subject of much empirical and theoretical study^23^. Here we used nearly 2,000 forest inventory plots to estimate the total number of tree species for the entire Amazonian forest and compare it to earlier estimates^1,4,12,22^. Our first finding was that despite the updated taxonomy (i.e. reduction of almost 10% of the species) and the addition of nearly 800 plots, the estimates of total species richness by each method were fairly similar. Updated point estimates using LSE and LS had a difference of -5% and 1.3% with our previous estimates^1^, and the updated estimate using the TNB had a difference of only 0.9% with previous estimates^12^. Such a small variation was the outcome of the well-known increase of number of species recorded as new plots were added to the sample, compensating the reduction of species by the taxonomic updating. Both processes affected the abundance distributions in the same way, as most of the species removed from the 2013 data set and most of the species added in the 2019 data set are among the less abundant ones.

Therefore, the updates affected the evenness of the abundance distributions in the samples, mainly by changing the number and relative abundances of rare species. These changes directly affect the estimates of species richness by LS and TNB, which rely on the shape of the distribution of abundances in the sample. Moreover, these changes also affect LSE and ABC estimates indirectly, because these methods use the distribution of total population sizes, which in turn depends on the relative abundances of species in the sample and on the occurrence of species across plots.

Also, we also updated the total area of Amazonian forest^22^ (see methods), causing a reduction of 17% in the estimated total number of trees. This is an additional cause of the decrease in the 2013 estimates of species richness, as all methods we used upscale some abundance distribution to the total size of the community.

The aggregation of species forms another important aspect of species richness estimation^24^, and as we have shown here, this greatly influences species estimations. All estimators make assumptions about the probability of occupancy, which is then used to estimate the expected number of species recorded if the whole area would be sampled. The occupancy is affected by the distribution of individuals across plots, and is higher under random distribution of species than if species are aggregated (as more aggregation leads to less occupied plots, under the same mean density per plot). Thus, if is there conspecific aggregation the assumption of random distribution underestimates the real number of species, as our simulations have shown. This effect was even greater in our data set because the rarer the species, the more aggregated it was. This positive relationship between abundance and aggregation was found at local scales in tropical forests^19^. For ATDN data sets this pattern results from larger scale processes, such as environmental differences and dispersal limitation among plots, and is in line with the hyperdominance of a few, abundant species in Amazon^1^, and also with core-satellite hypotheses^25,26^. The relationship is linear in log-log scale, which means that the degree of aggregation (expressed by the inverse of parameter k in Eq. S8, Fig. S8) increases by a power function with the mean density of species. Thus, the expected occupancy probability of unrecorded species drops quickly as more plots are added to the sample and the more abundant species are recorded, making the collectors curve rise slower than expected by random distribution of species. Thus, completing a list of species for Amazon is a long-term task that depends of increasing effort in data gathering. Such efforts include the expansion of plot-based inventory networks, such as ATDN, the increase of collecting efforts in existing plots, make fertile material available in herbaria for taxonomic experts, support taxonomic work, exchange information between field ecologists and taxonomist^27^. It also depends on other strategies to optimize the chances of adding new records to the list of Amazonian tree species, such as quick species assessments in target areas, and compilation of curated herbarium data^7,28^.

The main source of variation of richness estimates were the estimation methods. Therefore, which method should we trust? Considering that the current number of tree species recorded for the Amazon region is a little over 10,000^8^ methods that provide uncorrected estimates much below this value are definitely inappropriate for estimating the total richness of the Amazonian tree flora (PLN, 5649). In fact, PLN had poor support from all data sets. Using non-parametric methods would also result in estimates between 5,000 and 6,000, leading to rejection. Non-parametric methods are based on assumptions that do not meet sampling with tree inventory plots in large areas such as the Amazon^6^, and tend to hugely underestimate richness when sampling is below 40%^6,18^ – sampling here was 0.00035%. We have no good explanation why the PLN had such poor support from the data, however.

TNB provided more accurate estimates of species richness than LS when applied to simulated samples with aggregation taken from a lognormal distribution with 5,000 species, and also when applied to empirical data sets^12^. We added to these results a comparison of LS and TNB with information theoretical criteria, showing that TNB had better support from the distribution of abundances in the samples. Nevertheless, our more comprehensive simulations (simulated samples from LS and TNB, with and without clumping and with species richness ranging from 10,000 to 20,000) showed that the degree of clumping found in our data sets can make TNB fit better even if the sampled distribution is a LS. Although both LS and TNB models assume random sampling (the mean field assumption sensu^12^), TNB has an additional scaling parameter that allows a better fit to different types shapes of SADs^12^, and thus can accommodate better the increased dominance in abundance samples caused by conspecific clumping. Such versatility^12^ comes at the cost of a selection bias against LS when the species are not distributed randomly at the scale of sampling units. Indeed, the distribution of total population sizes were best approximated by simulated samples of LS metacommunities than by samples from TNB. Moreover, when fitted to samples from LS, TNB provided estimates with low precision. Combined, our current and previous^12^ simulations suggest that benefits of using TNB to estimate species richness and as a model for metacommunity SADs still needs further investigation.

Looking towards the other alternatives (LSE: 16,741; ABC: 14,941; LS: 14,996, Fig. 2), the choice of the best estimate for the entire Amazon still is not an easy one, because differences between them are not trivial in numbers. Differences in thousands of species are around 10% for the Amazon tree species richness but they are bigger than the tree diversity of North America (680^29^) or Europe (250). Recent reviews with simulated data support both LS^6^ and LSE^30^ as accurate models to upscale SADs from samples to metacommunities. The effectivity of ABC for inferring community parameters has not been evaluated systematically so far (but see^31^ for a particular example). Nevertheless, ABC is unique among methods we used because it estimates richness directly from the simulations that allow different degrees of dispersion among species. As detailed above, these simulations were also used to correct ad hoc the bias of the other methods.

Given all the considerations outlined above, we argue here that using these different methods and taking into account their pros and cons, the most reliable estimate is that of a weighted average providing an upper and lower bound of the estimated species richness.

Because the most up-to-date dataset already contains 5,027 tree species and considering that the 1,946 plots constitute a very small sample of the complete Amazonian forest (0.00035%), estimates close to that number are clearly an underestimate. Any trustable estimate should at least be more than the ~10,000 tree species already collected in Amazonian forests^8^. The LS, LSE and ABC estimations showed a wide range of richness, so which species estimate is most believable? As we cannot make a definite choice for any of the parametric methods (after all, the real number of species is not known), we suggest that the most probable estimate for the number of tree species in Amazonia is 15,874 species, based on a logseries with conspecific aggregation. We have shown that increasing the plot effort two or ten-fold will not contain more than 50% of the total estimated richness. A ten-fold increase of the current number of ~1-ha plots would still represent only 0.0035% of the forest area of Amazonia. Significantly increasing either the plot effort or implementing new, more intensive local sampling schemes (e.g.^15^) seem to be inconceivable in any near future. Therefore, the number of tree species in Amazonia would remain as much an estimate as it is now and even a rather intensive well planned collecting campaign^7,28^ will only resolve part of the “dark diversity” of trees in Amazonia.

## Methods

To delineate the surface area for estimation, we created a base map of Amazonia, the borders of which were the same as those in our earlier estimate^1^. Following^8,22^, we gridded this landscape^32^ into 0.1-degree grid cells (01DGC) and eliminated all 01DGCs that were more than 50% open water^32^, non-forest vegetation such as open wetlands or savannahs^33,34^, or>500 m elevation^35^. We quantified the area of all individual 1-degree grid cells (1DGCs), which varies with latitude due to distance from the equator (~124 km^2^ at the equator, ~106 km^2^ at 14° S, and ~120 km^2^ at 8° N). The final forest map consisted of 46,986 01DGCs, totalling 5.79 million km^2^ of forest area (Supplementary Fig. 10). This is considered the original extent of Amazonian forests. We made no correction for deforestation^22^, as all plots are in undisturbed forest. Therefore, predictions of the population sizes and species richness estimates relate to the original Amazonian forest cover.

### Tree density

Our tree-inventory data are from the ATDN network^1,9^. March 2019, the ATDN network comprised of 1,946 (1,774 1-ha, 146 < 0.5 ha, 26 > 2 ha) tree inventory plots with information on species composition and abundances, and an additional set of 274 plots for which only tree density is known. These forest plots are scattered throughout Amazonia (Supplementary Fig. 11) and located in all the major forest-soil combinations^1^. The total plot area of the 1,946 plots with composition data is 2,042 ha. Our composition plot sample thus amounts to 0.00035% of the Amazon forest area.

The methods we used to estimate the density of trees ≥10 cm dbh, species population sizes, and distribution are similar to those of^1,22^. From the 2,220 (= 1,946 + 274) plots with known tree density we removed outlier plots with less than 200 (59 plots) and plots with over 900 stems (105 plots) (Supplementary Fig. 12). We constructed a loess regression model for tree density (stems ha^−1^) based on the observed tree density in the remaining 2,056 plots (using latitude, longitude, and their interaction as independent variables). The span was set at 0.5 to yield a relatively smooth average. The model was used to estimate average tree density (D_1DGC_, stem/ha) in each 1-degree grid cell (1DGC). This average density was then multiplied by the total forested area of each DGC (see above) to obtain the total number of expected trees in the DGC.

### Population sizes and species distributions

Analyses of tree species composition were carried out using the 1,946 plots having species composition. Species synonymy was updated following^4,8^, which resulted in a reduction of almost 10% of the species observed in our sample in comparison to the 2013 version of the ATDN database. Species with a “*cf*.” identification were accepted as belonging to the named species, while those with “*aff*.” were tabulated at the genus level and therefore removed from the analysis.

While we assume that identification error is within acceptable limits for common species (see discussion in^1^), plots vary in the proportion of individuals identified to species. Plots in which this proportion is 75% or greater (1,695 plots) were used for the population estimates of all species. Additionally, for each species we added those plots of the remaining 251 in which the species was identified positively, assuming that where species are known, they usually are locally common and where they are unknown, they are locally rare. In doing so, we assume that this does not add too many false positives. At the same time, we avoid adding too many false negatives, when using the plots with poor resolution in species identifications. Therefore, the number of plots used for the calculation of the population size differed across species.

The number of trees belonging to each species in the 1DGC was estimated following^1,22^. Abundances of all valid species were converted to relative abundances (fractions) for each plot: *RA*_*i*_ = *n*_*i*_ */N*_*t*_, where *n*_*i*_ equals the number of individuals of species *i* and *N*_*t*_ the total number of trees in the plot (including unidentified trees)^1^. For all species with a valid name in the 1,946 plots, we constructed an inverse distance weighting (IDW) model for *RAi*, with a distance-decay power of 2, a maximum number of plots used for each local estimation of 150, and a maximum distance parameter of 4 degrees. The number of individuals of species *i* in a given 1DGC was then simply calculated as the total number of trees in the 1DGC (D_1DGC_) multiplied by the fraction of the species *i* for that same 1DGC.

### Amazonian tree-species richness

We provide estimates for three different versions of the tree inventory data: the 2013 version which contained less plots (1,170) and used an old taxonomy (hereafter the 2013 dataset), the updated 2013 version which also contains less plots (1,162) but uses the updated taxonomy (the updated 2013 dataset), and the 2019 version which includes all 1,946 plots and uses the updated taxonomy (the 2019 dataset). For each version of the data (Table S1), we estimated species richness for the original forest area of Amazonia using two different approaches: (i) extrapolation from the distribution of estimated total population sizes^1^; and (ii) parametric methods, using the species abundances recorded in the sampled plots (Fig. 1, right panels). The parametric methods include the fit of the logseries (LS)^36^, the Poisson lognormal (PLN)^13,37^, and the negative binomial (NB)^12^ to the empirical SAD of the plot data (Fig. 1., right panels) and the upscaling of the model fits to the total area of Amazonia. A summary of each estimation method follows below (more details are available in the supplementary material).

#### Logseries extension (LSE)

Using the population sizes of all Amazonian trees as in refs. ^1,22^ (and as outlined above), this method expands the species abundance distribution of these population sizes down to the species with only 1 individual. Under the assumption of an underlying logseries SAD (see description of logseries below), such expansion is well approximated by a linear extrapolation from the central part of the empirical SAD^1,10^. We calculated bootstrap confidence intervals of the total number of species estimated by LSE estimates using the standard deviations of the estimated population sizes, based on 500 bootstraps of the plot data (supplementary info page 5).

#### Logseries analytical (LS)

The logseries was among the first attempts to mathematically describe the relationship between the number of species and number of individuals in random samples from ecological communities^36^ as:

1 $${\rm{S}}={\rm{\alpha }}\,\mathrm{ln}(1+{\rm{N}}/{\rm{\alpha }})$$

where α is the single free parameter of the logseries, which can be estimated from the distribution of species abundances in a sample. We fitted the logseries to the empirical SAD and then used the estimated value of α to estimate number of species predicted by Eq. (1) using N as the total number of trees estimated for the whole Amazon (supplementary info page 5).

### Zero-truncated poisson lognormal (PLN)

Like the logseries, the PLN was developed based on the sampling theory of SADs^24,38^. It assumes that the observed SAD can be described as a Poisson sample of a regional SAD that follows a lognormal distribution, which is approximated by the ‘veil line’ truncation of the lognormal^39^. The PLN fitted to empirical SADs is truncated at zero, as species with no individuals recorded in the sample are unknown. As any zero-truncated distribution, the PLN fitted to an empirical SAD allows the calculation of the proportion of species that have not been sampled, thus allowing to estimate the total number of species in the sampled community^13,37^ (supplementary info page 6).

### Zero-Truncated Negative binomial (TNB)

The TNB also results from a Poisson sample, yet from a Gamma distribution. It has two free parameters, *r* and ξ_p_, and at the limit *r* -> 0 the TNB converges to the LS^12,24,36,38^. After fitting the TNB to the empirical SAD, we used the sampling intensity *p* (the proportion of all individuals included in the sample or the proportion of total area covered by the sample) to estimate the number of species in the entire Amazon (*S*) using the following equation^12^:

2 $${\rm{S}}={{\rm{S}}}_{{\rm{p}}}{(1-(1-{\rm{\xi }}))}^{r}/(1-{(1-{{\rm{\xi }}}_{{\rm{p}}})}^{{\rm{r}}})$$

$${\rm{where,}}\,{\rm{\xi }}={{\rm{\xi }}}_{{\rm{p}}}/(p+(1-p)\,{{\rm{\xi }}}_{{\rm{p}}}).$$

The fit of the LS, PLN and TNB models to the empirical SAD, was performed using maximum likelihood techniques with functions from the ‘*sads’* R Package^40^ (supplementary info page 13). The support that each data set provide for each of these three competing models was gauged by Akaike Information Criterion (AIC) and also by the Bayesian Information Criterion (BIC). (supplementary info page 6).

### Adding conspecific aggregation

Assuming that the trees in our plots constitute a sample from the unknown regional Amazonian SAD, we tested if sampling from this theoretical SAD could provide accurate estimates of the calculated population sizes and total species richness. We performed this assessment by upscaling the LS, PLN and TNB for the entire Amazonia. We then simulated 1,946 random draws of 1-ha plots from regional SADs generated by these models, with and without conspecific aggregation. In both cases, we assumed that the expected abundance of each species in each plot was its mean density (ha^-1^) estimated for the total area of Amazonia. As in other theoretical studies^24^, we used a Poisson distribution to simulate samples of randomly distributed species and a Negative Binomial distribution to simulate conspecific aggregation (not to be confounded with the TNB model described above).

We simulated the sampling of 1,946 1-ha samples from unknown regional SADs ranging from a total of 10,000 to 20,000 species. For each simulation and each estimation method (LSE, LS, PLN or TNB), we applied the same methods described above to estimate the total species richness. We then estimated the bias of each method, defined here as the mean difference between the known values of species richness in the theoretical SAD and the richness estimated by each method. We used the estimated bias of each method to calculate their bias-corrected species richness.

For simulations with conspecific aggregation, we allowed species to have different degrees of spatial aggregation, that is, different values of the dispersion parameter *k* of the negative binomial distribution. We obtained species-specific values of *k* based on the relationship between the estimated the values of *k* and the mean density of each species observed in the 1,946 plots (supplementary material page 7).

Moreover, we assessed which combination of theoretical SAD (LSE, LS, PLN or TNB) and sampling scheme (random or aggregated) best approximated the distribution of estimated population sizes. We evaluated the performance of each combination by comparing their proportion among of the set of simulations that best approximated the distributions of calculated population sizes, using Approximate Bayesian Computation (ABC)^41,42^, which was also used to build the credible intervals for the species richness estimates (supplementary material page 9).

### Influence of increasing sample size

We also used simulations to estimate the number of additional species that would be recorded if we increased the sample size (i.e., adding new 1-ha plots). We simulated the sampling procedure from a logseries with conspecific clumping, as described above. This logseries had a total number of trees equal to the estimated number of trees from the ATDN data set from 2019 (estimate presented in the results). We simulated samples with sample size varying between 1,046 and 19,460 1-ha plots. This range of sample sizes correspond to 90% of the number of plots in the 2013 updated data set (1,162 plots) to ten times the number the plots in the 2019 data set (19,460 plots). For each sample size we repeated the simulations 100 times and then calculated the mean number of species recorded in the simulated sample. The 100 simulations were also used to calculate the lower (5%) and upper bounds (95%) for each sample size.

## Supplementary information

Supplementary Material: Biased-corrected richness estimates for the Amazonian tree flora.

Supplementary Dataset 1.
