# Supplementary material for: Biased-corrected richness estimates for the Amazonian tree flora
Source: Sci Rep. 2020 Jun 23;10:10130. doi: 10.1038/s41598-020-66686-3 (PMC7311553; doi:10.1038/s41598-020-66686-3)
Supplement: Supplementary file 1 — Supplementary Material: Biased-corrected richness estimates for the Amazonian tree flora. [file 41598_2020_66686_MOESM1_ESM.pdf]

**Supplementary Material: Biased-corrected richness estimates for the Amazonian tree flora**

Hans ter Steege,1,2* Paulo I. Prado,3* Renato F. Lima,1,3 Edwin Pos,4,1 Luiz de Souza Coelho,5 Diogenes de Andrade Lima Filho,5 Rafael P. Salomão,6,7 Iêda Leão Amaral,5 Francisca Dionízia de Almeida Matos,5 Carolina V. Castilho,8 Oliver L. Phillips,9 Juan Ernesto Guevara,10,11 Marcelo de Jesus Veiga Carim,12 Dairon Cárdenas López,13 William E. Magnusson,14 Florian Wittmann,15,16 Maria Pires Martins,5 Daniel Sabatier,17 Mariana Victória Irume,5 José Renan da Silva Guimarães,12 Jean-François Molino,17 Olaf S. Bánki,1 Maria Teresa Fernandez Piedade,18 Nigel C.A. Pitman,19 José Ferreira Ramos,5 Abel Monteagudo Mendoza,20 Eduardo Martins Venticinque,21 Bruno Garcia Luize,22 Percy Núñez Vargas,23 Thiago Sanna Freire Silva,24 Evlyn Márcia Moraes de Leão Novo,25 Neidiane Farias Costa Reis,26 John Terborgh,27,28 Angelo Gilberto Manzatto,29 Katia Regina Casula,26 Euridice N. Honorio Coronado,30,9 Juan Carlos Montero,31,5 Alvaro Duque,32 Flávia R.C. Costa,5 Nicolás Castaño Arboleda,13 Jochen Schöngart,18 Charles Eugene Zartman,5 Timothy J. Killeen,33 Beatriz S. Marimon,34 Ben Hur Marimon-Junior,34 Rodolfo Vasquez,20 Bonifacio Mostacedo,35 Layon O. Demarchi,18 Ted R. Feldpausch,36,9 Julien Engel,17,37 Pascal Petronelli,38 Chris Baraloto,37 Rafael L. Assis,39 Hernán Castellanos,40 Marcelo Fragomeni Simon,41 Marcelo Brilhante de Medeiros,41 Adriano Quaresma,18 Susan G.W. Laurance,28 Lorena M. Rincón,5 Ana Andrade,42 Thaiane R. Sousa,5 José Luís Camargo,42 Juliana Schietti,5 William F. Laurance,28 Helder Lima de Queiroz,43 Henrique Eduardo Mendonça Nascimento,5 Maria Aparecida Lopes,44 Emanuelle de Sousa Farias,45,46 José Leonardo Lima Magalhães,47,48 Roel Brienen,9 Gerardo A. Aymard C.,49 Juan David Cardenas Revilla,5 Ima Célia Guimarães Vieira,7 Bruno Barçante Ladvocat Cintra,50 Pablo R. Stevenson,51 Yuri Oliveira Feitosa,52 Joost F. Duivenvoorden,53 Hugo F. Mogollón,54 Alejandro Araujo-Murakami,55 Leandro Valle Ferreira,7 José Rafael Lozada,56 James A. Comiskey,57,58 José Julio de Toledo,59 Gabriel Damasco,60 Nállarett Dávila,61 Aline Lopes,18,62 Roosevelt García-Villacorta,63,64 Freddie Draper,65,37 Alberto Vicentini,14 Fernando Cornejo Valverde,66 Jon Lloyd,67 Vitor H.F. Gomes,68,69 David Neill,70 Alfonso Alonso,58 Francisco Dallmeier,58 Fernanda Coelho de Souza,14,9 Rogerio Gribel,5 Luzmila Arroyo,55 Fernanda Antunes Carvalho,14,71 Daniel Praia Portela de Aguiar,18 Dário Dantas do Amaral,7 Marcelo Petratti Pansonato,5,3 Kenneth J. Feeley,72,73 Erika Berenguer,74,75 Paul V.A. Fine,60 Marcelino Carneiro Guedes,76 Jos Barlow,75 Joice Ferreira,48 Boris Villa,77 Maria Cristina Peñuela Mora,78 Eliana M. Jimenez,79 Juan Carlos Licona,31 Carlos Cerón,80 Raquel Thomas,81 Paul Maas,1 Marcos Silveira,82 Terry W. Henkel,83 Juliana Stropp,84 Marcos Ríos Paredes,85 Kyle G. Dexter,86,87 Doug Daly,88 Tim R. Baker,9 Isau Huamantupa-Chuquimaco,23 William Milliken,89 Toby Pennington,36,87 J. Sebastián Tello,90 José Luis Marcelo Pena,91 Carlos A. Peres,92 Bente Klitgaard,93 Alfredo Fuentes,94,90 Miles R. Silman,95 Anthony Di Fiore,96 Patricio von Hildebrand,97 Jerome Chave,98 Tinde R. van Andel,1,99 Renato Richard Hilário,59 Juan Fernando Phillips,100 Gonzalo Rivas-Torres,101,102 Janaína Costa Noronha,103 Adriana Prieto,104 Therany Gonzales,105 Rainiellen de Sá Carpanedo,103 George Pepe Gallardo Gonzales,85 Ricardo Zárate Gómez,106 Domingos de Jesus Rodrigues,103 Egleé L. Zent,107 Ademir R. Ruschel,48 Vincent Antoine Vos,108 Émile Fonty,109,17 André Braga Junqueira,110 Hilda Paulette Dávila Doza,85 Bruce Hoffman,111 Stanford Zent,107 Edelcilio Marques Barbosa,5 Yadvinder Malhi,112 Luiz Carlos de Matos Bonates,5 Ires Paula de Andrade Miranda,5 Natalino Silva,113 Flávia Rodrigues Barbosa,103 César I.A. Vela,114 Linder Felipe Mozombite Pinto,85 Agustín Rudas,104 Bianca Weiss Albuquerque,18 Maria Natalia Umaña,115 Yrma Andreina Carrero Márquez,56 Geertje van der Heijden,116 Kenneth R. Young,117 Milton Tirado,118 Diego F. Correa,51,119 Rodrigo Sierra,118 Janaina Barbosa Pedrosa Costa,76 Maira

Rocha,18 Emilio Vilanova Torre,120,121 Ophelia Wang,122 Alexandre A. Oliveira,3 Michelle Kalamandeen,9,123 Corine Vriesendorp,19 Hirma Ramirez-Angulo,120 Milena Holmgren,124 Marcelo Trindade Nascimento,125 David Galbraith,9 Bernardo Monteiro Flores,126 Veridiana Vizoni Scudeller,127 Angela Cano,51,128 Manuel Augusto Ahuite Reategui,129 Italo Mesones,60 Cláudia Baider,130,3 Casimiro Mendoza,131,132 Roderick Zagt,133 Ligia Estela Urrego Giraldo,32 Cid Ferreira,5 Daniel Villarroel,55 Reynaldo Linares-Palomino,58 William Farfan-Rios,134,90 William Farfan-Rios,23 Luisa Fernanda Casas,51 Sasha Cárdenas,51 Henrik Balslev,135 Armando Torres-Lezama,120 Miguel N. Alexiades,136 Karina Garcia-Cabrera,95 Luis Valenzuela Gamarra,20 Elvis H. Valderrama Sandoval,137,138 Freddy Ramirez Arevalo,138 Lionel Hernandez,40 Adeilza Felipe Sampaio,26 Susamar Pansini,26 Walter Palacios Cuenca,139 Edmar Almeida de Oliveira,34 Daniela Pauletto,140 Aurora Levesley,9 Karina Melgaço,9 Georgia Pickavance,9

*corresponding authors. Email: hans.tersteege@naturalis.nl; prado@ib.usp.br
A full list of affiliations appears at the end of the paper

## Contents

1. Summary statistics of the ATDN data sets .......... 4
2. Estimates of tree species richness in Amazon - methods .......... 5
   Parametric estimates from estimated population sizes .......... 5
   Parametric estimates from species abundances in the plots .......... 5
   Adding conspecific aggregation by simulation of plot samples .......... 7
   Simulations that best approximate the distribution of total population sizes .......... 9
   Using selected simulations to estimate bias of the species estimators .......... 9
3. Estimates of tree species richness in Amazon – results .......... 10
   Model fitting, model selection and estimating species richness: .......... 10
4. Supplementary Fig. 1-9 .......... 13
5. Supplementary tables .......... 23
6. ATDN 2019 data set (Supplementary Fig. 10, 11) .......... 27
7. Tree densities (Supplementary Fig. 12) .......... 29
8. References .......... 30

# 1. Summary statistics of the ATDN data sets

**Supplementary Table 1** – Summary statistics of the ATDN 2019 data set, compared to the previous versions of the data base. The number and pooled area of plots and the number of trees species and individuals do not include the plots for which only tree density is known (see main text).

| | **2013** | **2013 updated** | **2019** |
|---|---|---|---|
| **Total Amazon lowland forest area (ha)** | $6.99 \times 10^{8}$ | $5.79 \times 10^{8}$ | $5.79 \times 10^{8}$ |
| **Estimated total Nr of trees in Amazon** | $3.90 \times 10^{11}$ | $3.06 \times 10^{11}$ | $3.06 \times 10^{11}$ |
| **Nr of plots** | 1170 | 1162 | 1946 |
| **Pooled plot area (ha)** | 1080 | 1072 | 2042 |
| **Nr of sampled trees** | 639,939 | 633000 | 1,101,368 |
| **Nr of sampled trees identified** | 553,949 | 541,155 | 979,614 |
| **Nr of sampled species** | 4962 | 4531 | 5027 |

# 2. Estimates of tree species richness in Amazon - methods

## Parametric estimates from estimated population sizes

**Logseries expansion (LSE)**: This method estimates total species richness from a line that joins the 25% and the 75% quantiles of the rank-abundance plot of the whole community [1,2]. Assuming that at least the last part of this rank-abundance plot is well approximated by a logseries, the extension of this line down to the smallest possible abundance hits the x-axis at the value of the total species richness [1,2]. Therefore, the LSE estimator is simply the ratio between the intercept and the slope of the line that cross the central quantiles of the rank-abundance plot of the community [2,3].

We thus derived this line from the rank-abundance plots of the estimated population sizes of all Amazonian trees, as in refs [1,4]. The intercept and slope of this line were estimated from a simple linear regression fitted to the values within the central quantiles of abundances (25%-75%). As the total abundances of trees are estimates (see ref *6* and methods in the main text), we used a parametric bootstrap to apply the LSE method taking into account the estimation errors of species abundances. We calculated the bootstrap confidence intervals of the populations of all species based on 500 bootstraps of the plot data.

We then simulated new abundances for each species by drawing independent values from Gaussian distributions. For each species, these Gaussian distributions had mean and standard deviation equal to the estimated abundance of the species, and the standard deviation of the estimate abundances, respectively. For each set of simulated abundances, we then estimated species richness by LSE. The final point estimate of LSE species richness was the mean of the estimates obtained from the bootstrap samples. The range estimate was the empirical bootstrap 95% confidence interval (that is, the 2.5% and 97.5% quantiles of the bootstrap LSE estimates). We simulated 200 bootstrap samples for each data set (2013, 2013 updated and 2019).

**Approximate Bayesian Computation (ABC):** we used ABC to find the combination of sampling and regional species abundance distributions that best approximates the empirical distribution of population sizes. As detailed in the section "Simulations that best approximate the distribution of total population sizes", this method provides point and range estimates of the regional species richness.

## Parametric estimates from species abundances in the plots

**Logseries (LS)**: The logseries was among the first attempts to mathematically describe the relationship between the number of species and number of individuals in random samples from ecological communities [5]. The logseries is a limit case of the distribution of abundances observed in a Poisson sample (i.e. sampling process in an infinite population with random species distributions), taken from a regional SAD (i.e. the entire Amazon here) with a large number of species. It is formally described by:

$$\Phi_n = \frac{\alpha x^n}{n} \qquad \text{(Eq. 1)}$$

where: $\Phi_n$ is the number of species with n individuals in the sample; α is Fisher's α and x = N/(N + α) with N being the number of individuals in the total sample. The logseries is essentially a geometric summation, which builds up from the first term ($\Phi_1$), the singletons. The number of singletons is thus predictable in a logseries ($\Phi_1 = \alpha x$) and always is the largest class. As x approaches 1 for reasonably large samples, $\Phi_1$ asymptotically approaches α in such samples. Hence, we expect α from samples to quickly approach α of the total landscape (i.e. metacommunity), after which it will be practically independent of sample size. The original numerical recipe to calculate Fisher's α from the known number of individuals (N) and species (S) is to solve iteratively :

$$\alpha = \frac{S}{\ln(1+N/\alpha)} \qquad \text{(Eq. 2)}$$

which is equivalent to the maximum likelihood method to estimate α [6]. By rearranging equation 2 we have the original expression proposed by Fisher for the expected number of species in a log series for a given N number of individuals:

$$S = \alpha \ln\left(1+\frac{N}{\alpha}\right) \qquad \text{(Eq. 3)}$$

Which we then used to estimate the total number of tree species in Amazon for each data set, from the values of α estimated from the fit of logseries to the species abundances in the sample of plots and the total number of trees estimated for Amazon (see 'Tree density' in the main text).

**Zero-truncated Poisson lognormal (PLN):** Like the logseries, the PLN was developed based on sampling theory of SADs [7,8]. It assumes the observed SAD can be described as a Poisson sample of a regional SAD that follows a lognormal distribution, which is approximated by the 'veil line' truncation of the lognormal [9]. The Poisson-lognormal is the analytical solution for this sampling model, as Fisher's logseries is an analytical limit case for a Poisson-gamma (or negative binomial) distribution. The Poisson-lognormal has two parameters: $\sigma_p$, which corresponds to the parameter σ of the underlying lognormal (the standard deviation of the logarithm of species abundances), and $\mu_p$, which corresponds to the parameter μ of the lognormal (the mean of the logarithm of species abundances) plus the logarithm of sampling intensity:

$$\mu_p = \mu + \log(p) \qquad \text{(Eq. 4)}$$

The PLN fitted to empirical SADs is truncated at zero, as species with no individuals recorded in the sample are unknown. As any zero-truncated distribution, once fitted PLN allows the proportion of species that have not been sampled to be calculated, thus allowing to estimate the total number of species in the sampled community [10,11].

**Zero-Truncated Negative binomial (TNB)**: The TNB is widely used as a phenomenological model to fit count data with aggregation. It is the distribution that again results from a Poisson sample, yet from a Gamma distribution [8]. The TNB can also be derived from first principles based on simple birth-death-immigration stochastic dynamics [12-14]. It has two free parameters, *r* and $\xi_p$, and at the limit r -> 0 the TNB converges to the LS [5,7,8,12]. After fitting the TNB to data, we can use sampling intensity *p* (the proportion of all individuals included in the sample or the proportion of total area

covered by the sample) to estimate the number of species in the entire Amazon ($S$) using the following equation [12]:

$$S = S_p \frac{(1-(1-\xi))^r}{1-(1-\xi_p)^r} \qquad \text{(Eq. 5)}$$

where

$$\xi = \frac{\xi_p}{p+(1-p)\xi_p} \qquad \text{(Eq. 6)}$$

**Model fitting, model selection and estimating species richness:** For each dataset we fitted LS, PLN and TNB to the distribution of abundances of species in the pooled plots. To fit each model to these empirical SADs we used computational maximization of likelihood functions using the R package '*sads*' [13]. We then used information-based model selection to identify the model best supported by each data set, using Akaike's Information Criterion (AIC, [15]).

For each of the three SADs models fitted, the estimated total number of species in the sampled community is a monotonic function of the fitted parameters. Thus, the values of these functions at the lower and upper limit of the likelihood intervals of the model parameters provides a likelihood interval of the estimated species richness [16]. We adopted the log-likelihood ratio of 2 to estimate these intervals.

## Adding conspecific aggregation by simulation of plot samples

As detailed above, the estimators of species richness that we used assume a model for sampling, a model for the species abundance distributions sampled, or both. All those methods, except ABC, assume independent and constant probability of any individual be included in the sample, which is more tractable analytically. For trees or any sessile organism this assumption implies in random spatial distribution of all species at the scale of sampling units (plots in our case). Nevertheless, tropical trees in general show spatial aggregation at a wide range of scales, while the degree of clumping varies among species [17-19]. Therefore, sampling models that take into account species-specific clumping patterns might be a more accurate description of the abundance and richness patterns found in the ATDN data set. Conspecific aggregation is indeed well defined in the sampling theory of SADs [7], and its outcomes can be easily assessed with parametric computer simulations (e.g. [6]). We simulated samples of alternative abundance distributions with and without species-specific clumping to (i) identify which combination of sampling and SAD best approximates the distribution of population abundances in each data set; (ii) use this best-approximation model to estimate biases in the estimations of species richness.

We generated random draws of 1-ha plots from logseries, lognormal and truncated negative binomial distributions with varying number of species, but keeping all other parameters from the empirical data. The total number of individuals in each sampled distribution was fixed to the total

number of trees estimated for Amazon, and the sample sizes were fixed at the number of plots, in each data set. For the parameter σ of the lognormal and parameters r and ξ of the truncated negative binomial simulated distributions we used the values estimated by fitting these distributions to the empirical data, as described in the previous section. The parameter μ of the lognormal was calculated from the estimated parameter $\mu_p$ of the Poisson-lognormal fit to the empirical data and the total number of trees (equation 6). Fisher's α of the logseries was calculated from the total number of trees and the number of species in each simulation (equation 2).

We drew values of abundances of each species in each plot from a Poisson distribution to simulate samples with random dispersion of trees, and from a negative binomial distribution to simulate samples with species-specific clumping. Both sampling distributions have a parameter that corresponds to their mean, which in our case is the density of each species per hectare in the whole area sampled. This parameter was then set to the species abundances in the simulated regional SAD divided by the total area of Amazon adopted in each data set. The negative binomial has one additional parameter that expresses the degree of dispersion. We assigned a value of this dispersion parameter (k) from its relationship with species densities in the empirical data sets, as follows, for each data set:

1 – Estimate the empirical values of k from the observed occupancy of each species (details below);

2 – Fit a linear regression of the empirical values of k as function of the species density, in log scale (Supplementary Fig. 8);

3 – Use this linear regression to assign a value of the parameter k to each species in the simulated regional SAD: take the density of each species from the regional SAD, calculate the expected value of k from the regression and add a random Gaussian error with zero mean and standard deviation equal to the residual standard error of the regression.

To estimate the empirical values of k for each species, we used computer optimization to find the value of this parameter that minimizes the likelihood function:

$$L(k \mid \mu, N, f_0) = f_0 \ln(1-p) + (N - f_0) \ln p \qquad \text{(Eq. 7)}$$

where $f_0$ is the number of plots where the species was not recorded, N is the total of plots in the data set and p is the occupancy probability (the probability of recording the species in each plot) according to a negative binomial with parameters μ and k:

$$p = 1 - \left(\frac{k}{k+\mu}\right)^k \qquad \text{(Eq. 8)}$$

where μ is the expected value, or the mean, of the negative binomial distribution. The likelihood function *L* is conditioned on (μ , N, $f_0$ ) because these parameters are taken from the empirical data, including μ, which was set as the density by 1-ha plots of each species. The likelihood function assumes that the observed number of occupied plots by each species in each data set is a binomial variable with a constant probability of success p. Therefore, we did not take into account spatial autocorrelation of occupancies, but even such first-order approximation for the effect of species-

specific clumping proved to be a better model in comparison with the assumption of random dispersion (results in the main text and below).

## Simulations that best approximate the distribution of total population sizes

**Approximate Bayesian Computation (ABC):** ABC compares summary statistics calculated from the data and from models to estimate (approximate) posterior probabilities of models and their parameters, given the observed data. We used an approximate Bayesian computation (ABC, [20,21]) to find which combination of regional SAD (logseries, truncated negative binomial or lognormal) and sampling (with random or species-specific clumping) best approximated the total population sizes estimated from each data set. ABC compares summary statistics output by competing models (usually simulations) with those observed in a dataset to assign (approximate) posterior probabilities to each model. The observed values of the statistics are targets to which the results of each simulations are compared, by a standardized Euclidean distance. The proportion of simulations of each model that are below a given distance threshold estimates the posterior probability of the model given the data, and thus provides a Bayesian model-selection criterion based on computational simulations.

We applied ABC to estimate the posterior distribution of the number of species, assuming that the population sizes estimated from the data sets are an output of a sample of plots from a logseries regional SAD, with species-specific spatial clumping. This simulation model had the highest posterior probability among competing combinations of regional SADs and sampling models (see results in the main text and the details about the simulations and ABC in the section 'Adding conspecific aggregation by simulation of plot samples', above).

We used four target statistics from the distribution of estimated population sizes of trees in Amazon, in each data set: number of species, inverse of Simpson's index (that is, Simpson's species equivalents), and mean standard deviation of the log of abundances. The simulated estimated population sizes took into account the empirical estimation errors as follows:

1 – From the estimated population sizes estimated in each data set fit a linear regression of the standard deviations of the estimates ($s_{pop}$) as a function of the estimated values, in log scale (Supplementary Fig. 9);

2 – Use this linear regression to assign a value of $s_{pop}$ to each species in the simulated regional SAD: take the abundance of each species from the regional SAD, calculate the expected value of $s_{pop}$ from the regression and add a random Gaussian error with zero mean and standard deviation equal to the residual standard error of the regression.

3 – Simulate the estimation of population sizes of each species as the abundance of the species in the regional SADs plus a random Gaussian error with zero mean and standard deviation equal to $s_{pop}$.

## Using selected simulations to estimate bias of the species estimators

We ran 10,000 simulations of samples of logseries regional SADs with the number of individuals fixed at the estimated total number of trees in Amazon for each data set. The number of species

varied among simulations, and was drawn from a uniform distribution ranging from 10,000 to 20,000. We calculated four summary statistics of the distribution of estimated population sizes output by these simulations (number of recorded species, Simpson's species-equivalent, mean and standard deviation of the logarithm of population sizes). The posterior distributions of species richness were estimated by the local linear ABC algorithm [20,21], which had the smallest estimation errors among the options available in the '*abc*' R package [21]. We also checked the estimation errors of three tolerance thresholds (0.05, 0.025 and 0.01) and choose the highest threshold with the lowest estimation error up to 2 decimal points. The errors were estimated by 100 leave-one-out cross-validation tests [20], and were up to 0.003. The posterior sample sizes were 500 for the 2013 and 2019 data sets, and 100 for the 2013 updated data set.

The simulations described above return species abundances in the samples and also estimates of total populations of species, for samples of SADs with 10,000 to 20,000 species. Such output allows to calculate all estimators of species richness we used (section 'Estimates of tree species richness in Amazon') and to compare these estimates with the true species richness used in each simulation. We used this approach to estimate and correct biases of the estimates of species richness, assuming that ATDN data is best approximated by a sampling with conspecific clumping of a regional logseries, as was shown by ABC.

# 3. Estimates of tree species richness in Amazon – results

Supplementary Fig. 2 shows the point and range estimates of species richness by LSE for and the other methods described above. The LSE bootstrap point estimate for the 2013 data set was 15,196 species, which is below the value previously reported of 16,000 [1]. Therefore, the estimation error of total species abundances caused an overestimation of species richness by LSE, which is corrected by our new bootstrap approach. Moreover, the LSE bootstrap estimates described in this section were corrected for biases caused by conspecific clumping at the sampling scale, as detailed in the next section. These bias-corrected estimates are the figures reported in the main text, and are compared to the uncorrected estimates in Supplementary Table 1 and Supplementary Fig. 4.

## Model fitting, model selection and estimating species richness:

Supplementary Table 2 shows the estimates of the parameters of LS, TNB and PLN fitted to each data set. For all data sets TNB was the best supported model, but LS had equal support from the 2013 data set according to AIC (Table 1 in the main text) or even the best support according to BIC (Table S6). PLN had virtually no support, and overestimated the abundance of common species and underestimated the number of rare species in the samples of all three data sets (Fig. 1 of the main text and Supplementary Fig. 1). TNB and LS showed the opposite trend, that is, overestimation of the number of rare species and underestimation of the abundances of most common species (Fig. 1 of the main text and Supplementary Fig. 1). The point and range estimates species richness by LS and TNB for each dataset are in Supplementary Fig. 2. Supplementary Fig. 4 and Supplementary Table 2 compare these estimates with their bias-corrected values (details below). We did not calculate the species richness estimates by PLN as this model had no support from the data.

We simulated each combination of sampling and regional SAD as defined above 10,000 times for values of regional species richness drawn from a uniform distribution ranging from 10,000 to 20,000. In ABC this is the prior distribution of the unknown parameter, which we define as an uninformative distribution between a value slightly below the know number of species in Amazon and twice it, (which is also larger than any of our estimates, Supplementary Fig. 3). We used a rejection threshold of 0.01, which means that only the 1% of the simulations that resulted in the smallest Euclidean distances to the empirical values of the summary statistics were kept to build posterior distributions.

Cross-validation tests show that ABC has a higher probability to distinguish among simulations of different regional SADs than among simulations of clumped or random sampling of the same theoretical regional SAD (Tables S4-S6). Nevertheless, there is a good distinction of the sampling models when the simulated regional SAD is the logseries (match probabilities above 80%, Supplementary Tables 3-5). The simulations that sampled a logseries regional SAD with species-specific clumping had posterior probabilities of at least 0.71, followed by the simulations of random sampling of the logseries (Table 2 of the main text). The remaining simulations had posterior distributions lower than 0.01 (less than 1 / posterior sample size). The 2019 data set provided the best support for the hypothesis that the distribution of total abundances is best approximated by sampling with conspecific clumping of a logseries (posterior probability = 0.91, Table 2 of the main text).

If the estimates are unbiased, their values should lay along the equivalence (1:1) line in a plot of the simulated number of species as function of the estimated number of species. This is the case for LS estimates under random sampling of a regional logseries (blue points in the plots of the upper row of Supplementary Fig. 3a-c, upper right panels). This is not surprising, as these simulations fill all the assumptions of the LS estimator of species richness. However, with conspecific clumping the LS estimated richness underestimates the simulated number of species (red points in the same plots). Still the relationship between true and estimated species richness kept linear and the bias was constant in all simulations. We thus estimated bias-corrected values for LS with a linear regression of the species richness in the simulations as function of the richness estimated by LS. We fit this regression to the subset of simulations that had LS estimates within the empirical richness range estimate of each dataset. The lines of these regressions are depicted in red, in Supplementary Fig. 3a-c. The bias-corrected estimates were then the species richness predicted by this linear regression for the empirical point and range estimates. The same method was used to estimate the bias-corrected estimates of species richness for LSE (upper left and lower right panels in Supplementary Fig. 5a-c).

Only for TNB there was not a clear relationship between species richness and estimations (lower left panels in Supplementary Fig. 3a-c). Moreover, simulated samples from richness ranging from 8,000 to 20,000 provided TNB estimates within the empirical range estimates (grey areas in the plots), suggesting a low precision of the estimates obtained from the data sets. This result held for samples with and without conspecific clumping (red and blue dots in the in Supplementary Fig. 5a-c). We thus conservatively used all simulations that returned TNB estimates within the empirical TNB range estimates (that is, all points that fell within the grey area in the plot of Supplementary Fig. 5a-c) to estimate biases of TNB. We calculate the bias-corrected TNB point and range estimates as the mean and 2.5%-97.5% quantiles of the richness in these selected simulations. The wide range estimate of

bias-corrected TNB thus express its low precision in estimating species richness from samples of a logseries SAD.

Conspecific clumping caused negative bias in all estimates but at different degrees (Supplementary Fig. 4). Correction of these biases reduced the discrepancy among estimates (Fig. 2 in the main text, Supplementary Fig. 4, Supplementary Table 2).

We also used the simulations to evaluate biases in model selection of TNB and LS, the two best supported models for the abundance distributions in the samples. We fit both models to the simulated samples from regional logseries and truncated negative binomial SADs, and then performed a model selection with AIC and BIC, as we did for the empirical data (see 'Model fitting, model selection and estimating species richness', above). For random (Poisson) sampling, LS fit had a high (ca 0.8 for AIC and ca. 1.0 for BIC) and constant probability of being the best supported model by simulated samples of a logseries regional SAD (Supplementary Fig. 5, left panels, black dots). Nevertheless, this probability (or evidence weight) was virtually null for the simulated samples of a logseries with conspecific clumping (Supplementary Fig. 5, left panels, red dots).  The TNB model had an even high evidence weight when fit to simulated samples of a TNB regional SADs with more than 10,000 species, with or without conspecific clumping (Supplementary Fig. 5, right panels, black and red dots). Therefore, conspecific aggregation causes a strong selection bias towards the TNB model against LS in simulations that are compatible with our data sets. This result provides an explanation for the best support of TNB over LS by the empirical sampled abundances, while ABC picked samples with clumping of LS as the best approximation model for the distribution of total population sizes.

The mean and 2.5% - 97.5% quantiles of the posterior distributions returned by the ABC algorithm were used as the point estimate of species richness, and the limits of the range estimate of species richness, respectively. Fig. 2 in the main text and Supplementary Table 2 report these ABC estimates of species richness, for each data set. The bias correction for conspecific clumping was not necessary for this estimator, because it is based in a simulation that takes into account such clumping. Actually, the simulation used to correct this bias in the other estimators was the same used in the ABC richness estimates (see the section on simulated plot samples, below).

# 4. Supplementary Fig. 1-9

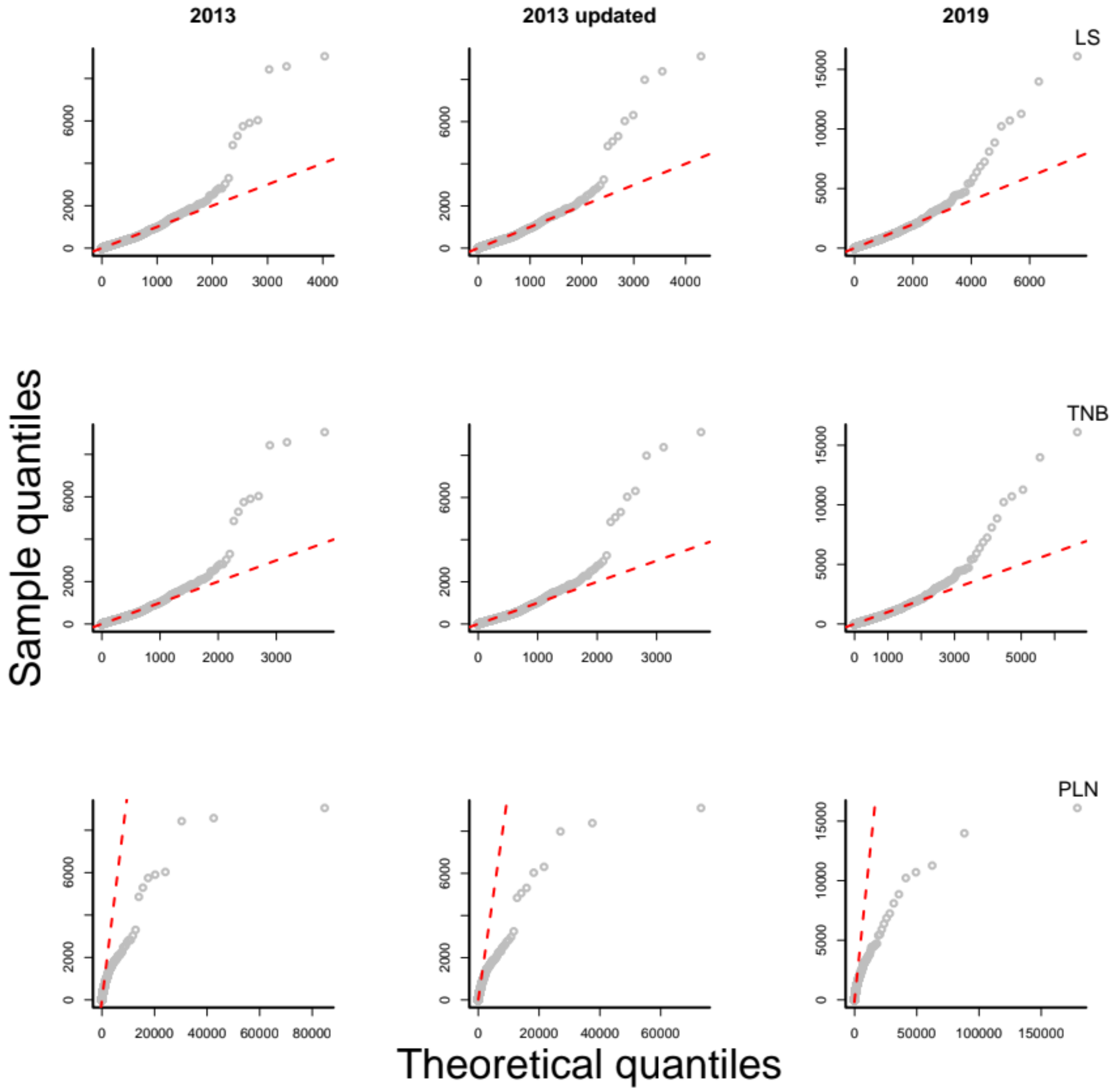


**Supplementary Fig. 1**. Quantile of the abundances in the samples in each of the data sets as a function of their values as predicted by the Logseries (LS), Truncated negative binomial (TNB) and Poisson lognormal (PLN) models fitted to each data set.

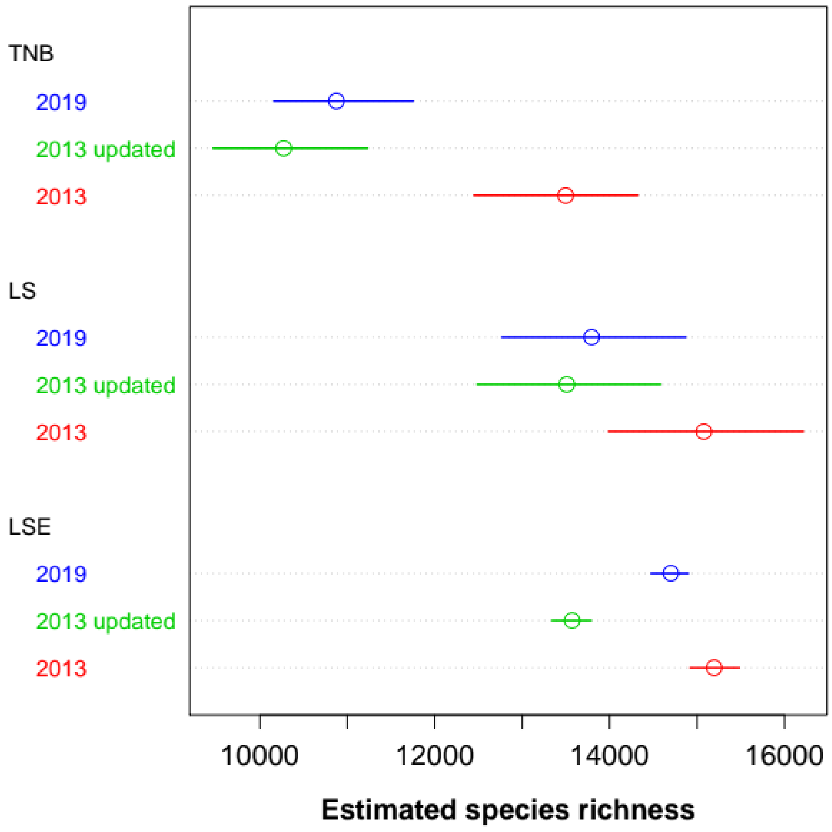


**Supplementary Fig. 2.** Original (uncorrected) estimates of species richness. Points are point-estimates and horizontal bars are range estimates (see Table S1 for details).

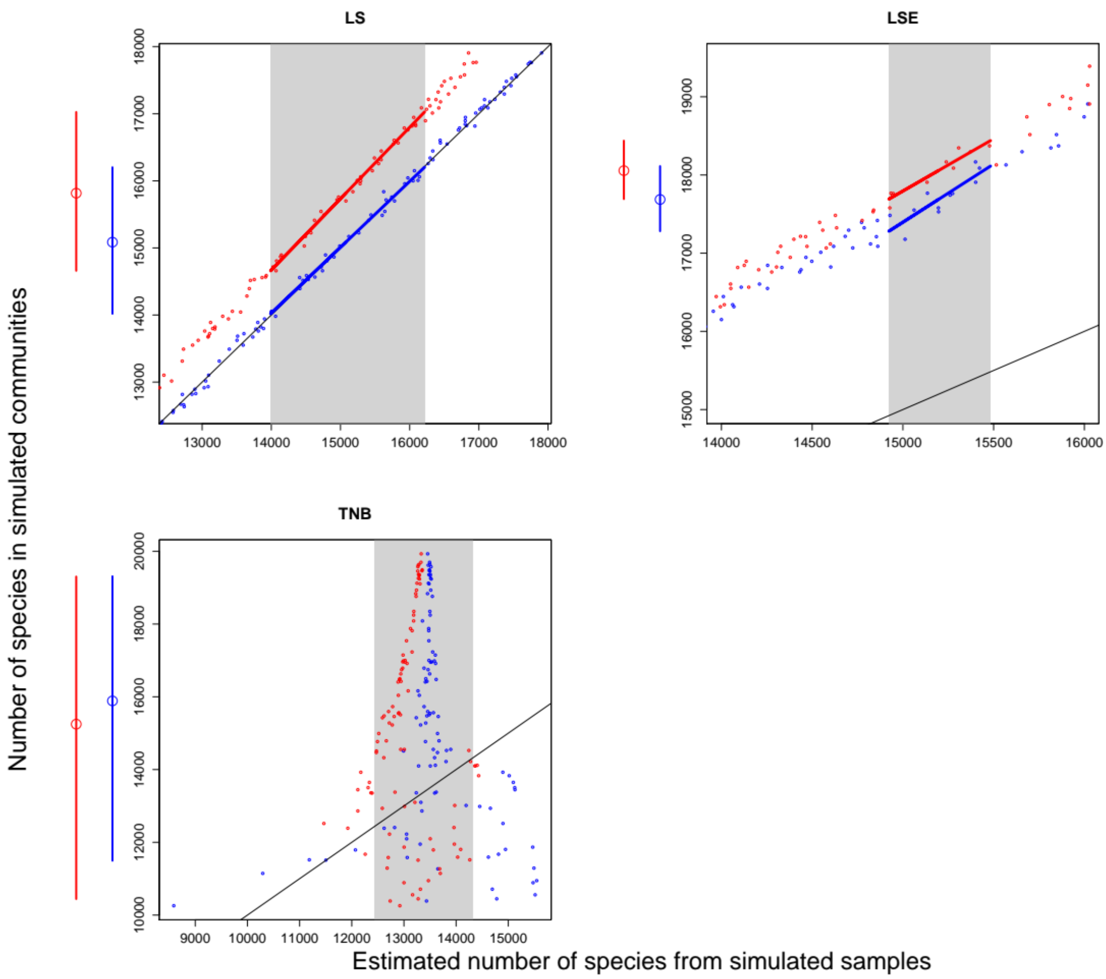


**Supplementary Fig. 3a**. Bias-correction of the estimations of total species richness for each method, for the 2013 data set. Each panel shows the true number of species in simulated communities (X) and the estimated species richness (Y) from simulated samples of the communities with the same properties of the empirical samples in the data set. The grey areas delimit the range estimate of the number of species estimated from empirical samples. The black line is the equivalence (1:1) line. Red and blue dots depict samples with and without conspecific clumping. Red and blue lines are the linear regression lines of richness in the community as a function of estimated richness, within the values of the empirical range (that is, a regression fitted to the points that fell within the grey area). The red and blue dots and lines at the left of each scatter plot shows the bias-corrected point and range estimates of species richness, estimated by the regressions (see text for details). For TNB there was not a linear relationship between true richness and the estimator, and then the bias-corrected estimates were calculated by the quantiles of the points in the grey area (see text for details). The estimation methods are: fitting of Logseries (LS) and Truncated Negative Binomial (TNB) to sample abundances, and Linear extension of the rank-abundance plots of estimated populations sizes (LSE). In all simulations the community has a Logseries SAD with the total number of individuals as the estimated from empirical data sets.

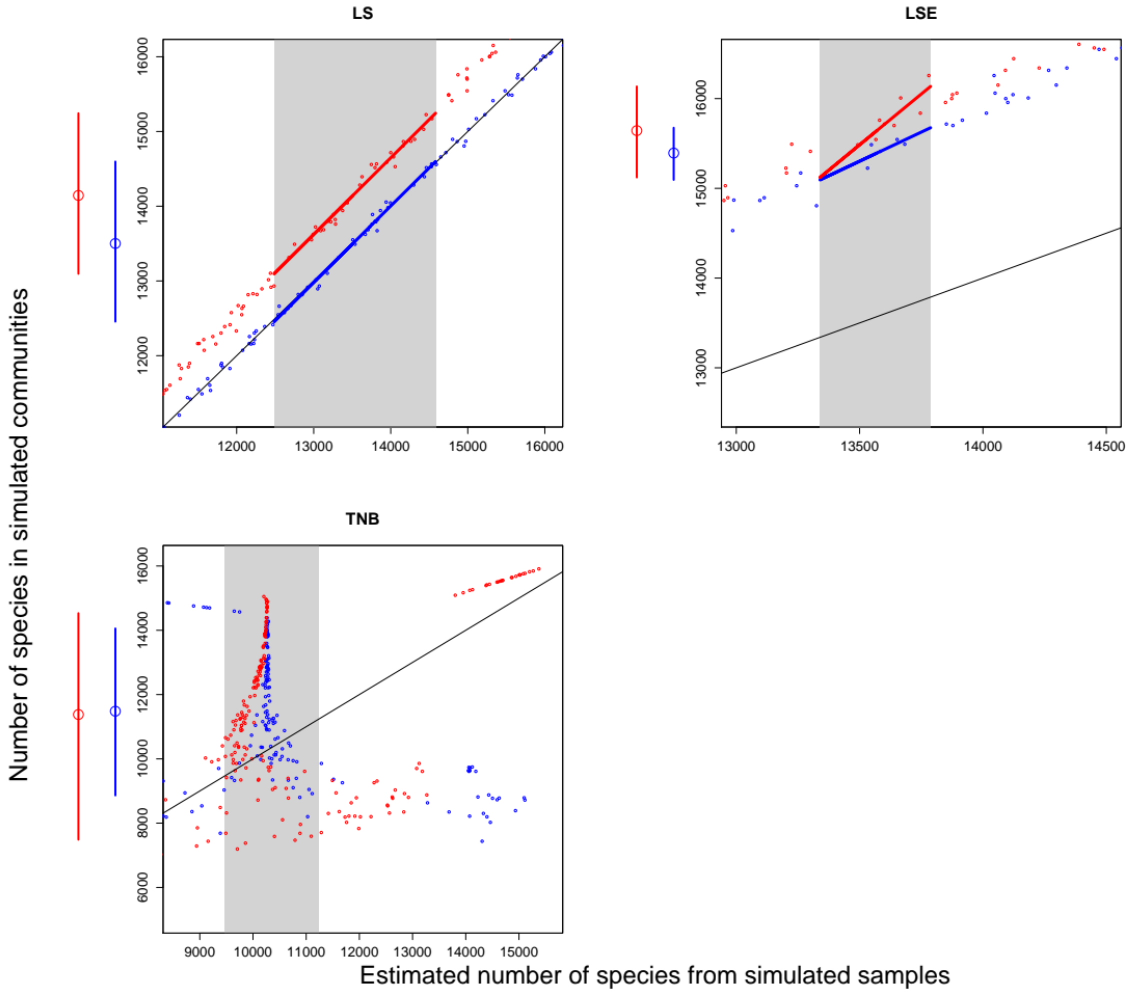


**Supplementary Fig. 3b**. Bias-correction of the estimations of total species richness for each method, for the updated 2013 data set.

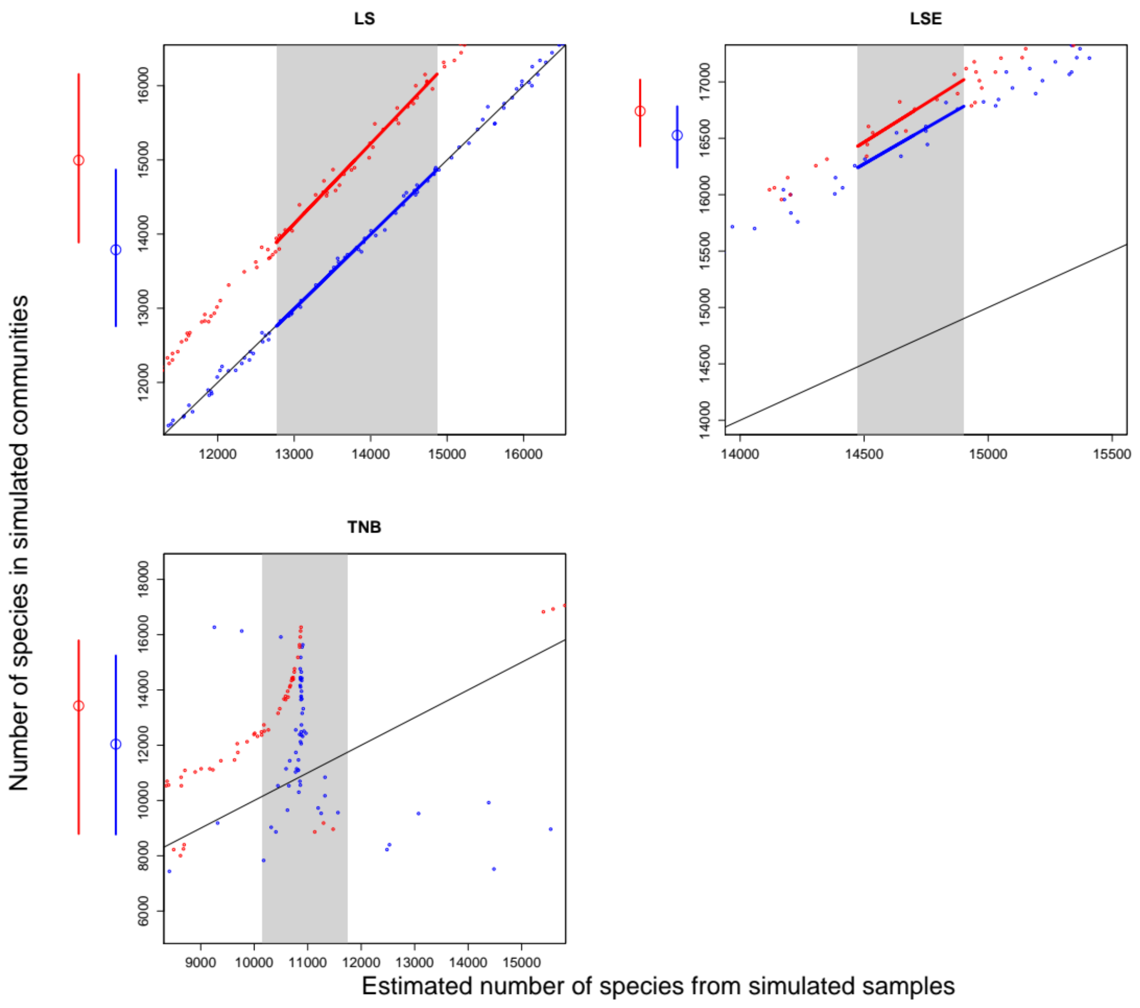


**Supplementary Fig. 3c**. Bias-correction of the estimations of total species richness for each method, for the updated 2019 data set.

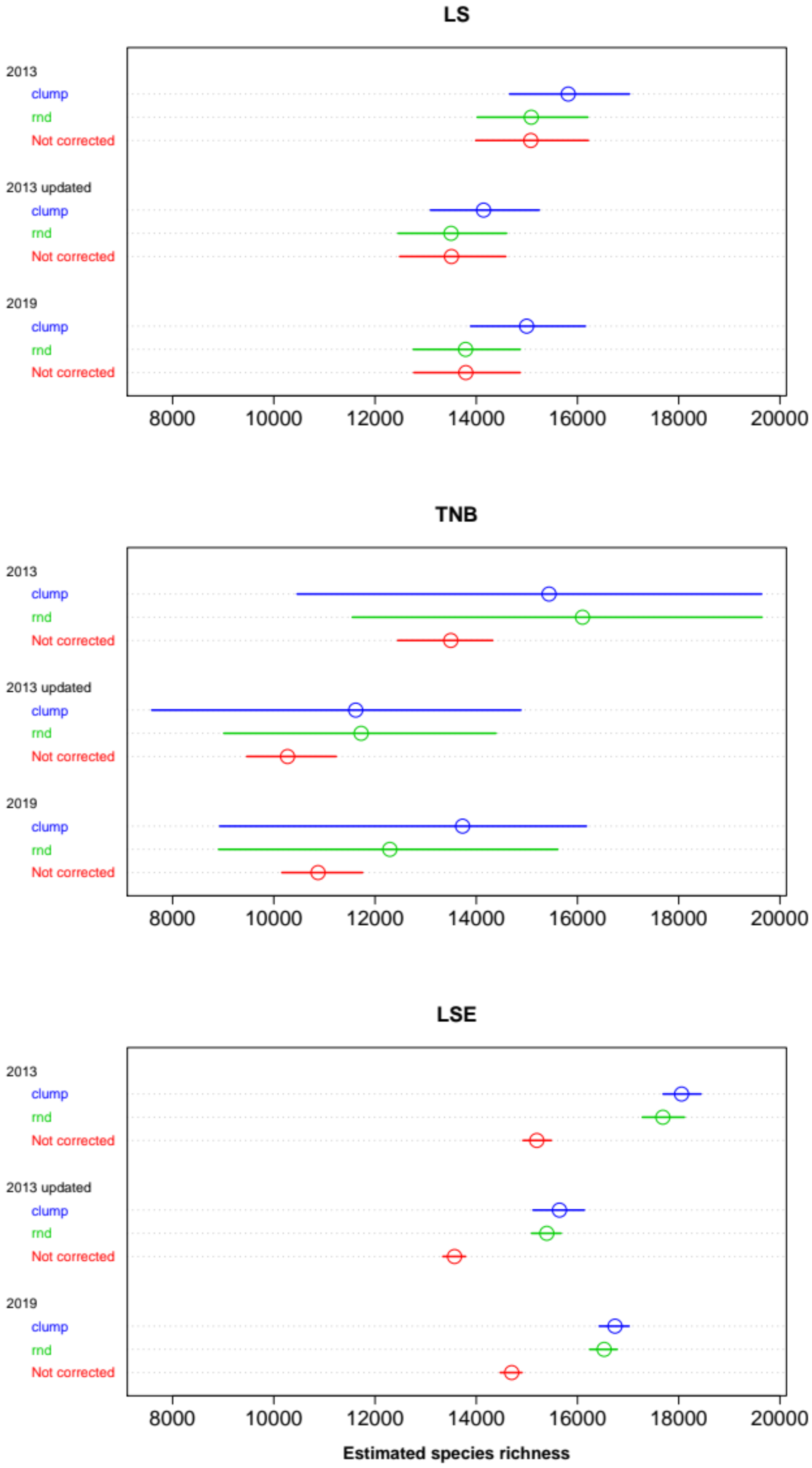


**Supplementary Fig. 4.** Original and bias-corrected estimates of species richness. Bias-correction assumed random or clumped sampling of a Log series regional SAD, with the same number of individuals estimated for the Amazon, and the same samples size of each data set (see methods in main text). Points are point-estimates and horizontal bars are range estimates (see Table S1 for details).

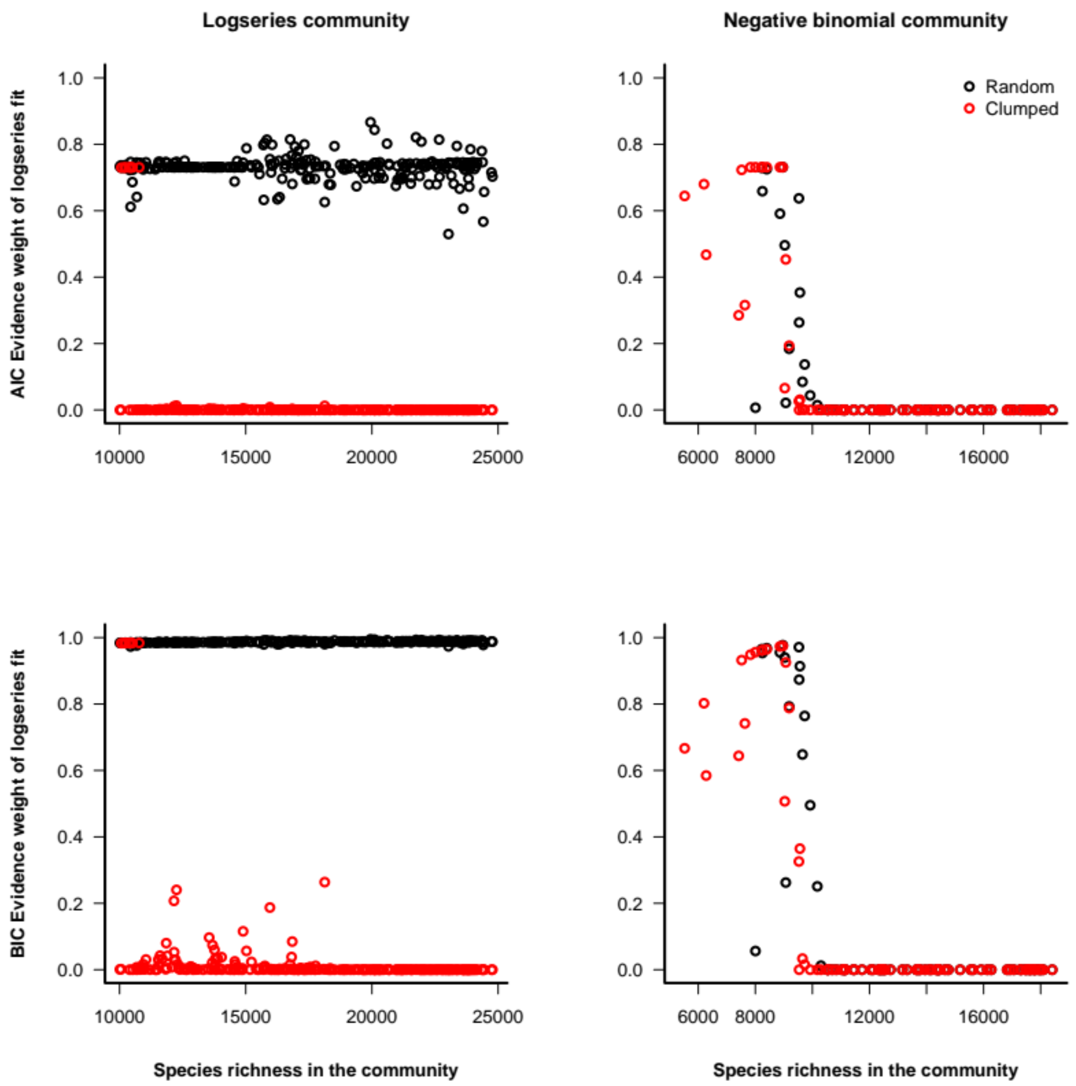


**Supplementary Fig. 5**. Akaike (upper row) and BIC (bottom row) evidence weights for the LS model against the TNB model in function of the total number of species in the community, fitted to simulated samples from a Logseries (right) and Truncated Negative Binomial (left) regional SAD. Samples were simulated with (red) and without (black) conspecific clumping. Evidence weights estimate the posterior probability of the LS fit to data to be better supported than the TNB fit, according to AIC or BIC.

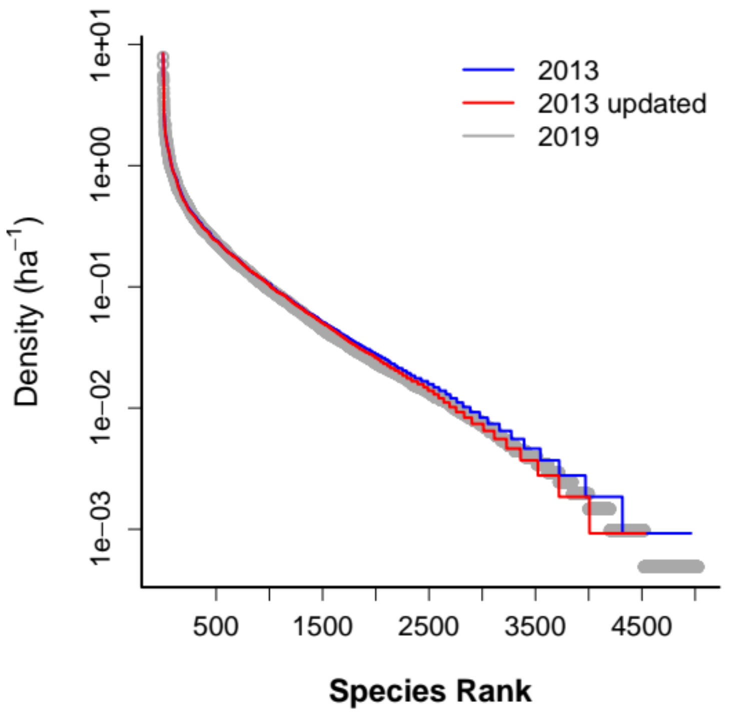


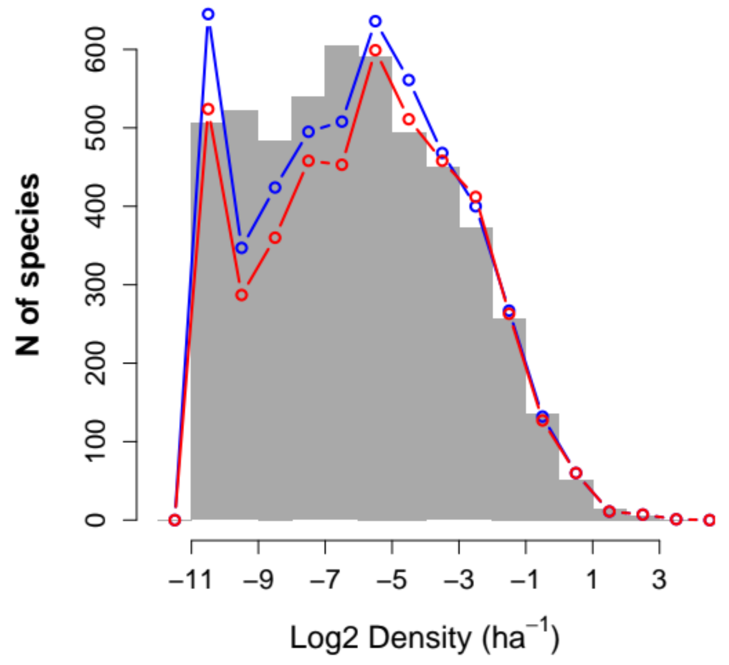


**Supplementary Fig. 6.** Species abundance distributions of trees in the plot sample for each data set. Abundances are expressed as density of recorded individuals per ha because the total area sampled is different among data sets (Table SX). **Upper panel:** rank-abundance plot. **Lower panel:** octave or Preston plot (histogram of the frequency of species in abundances classes in Log2 scale).

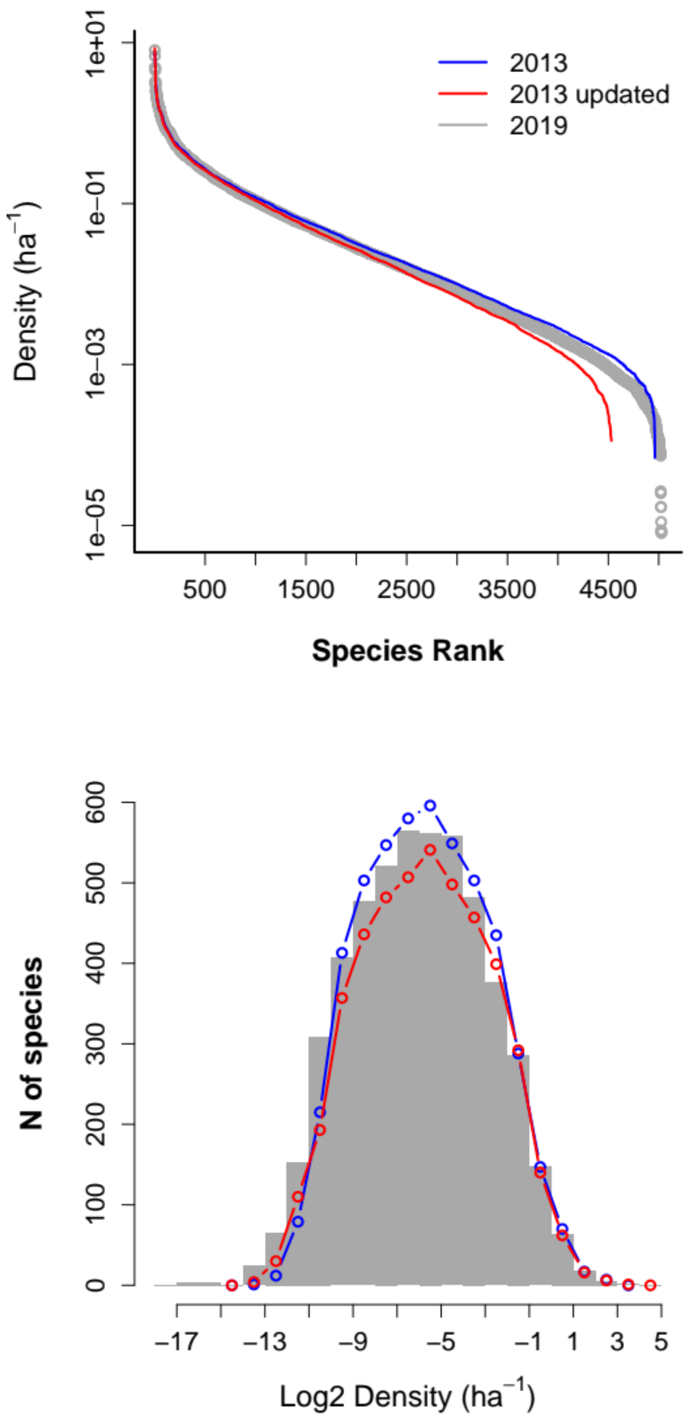


**Supplementary Fig. 7.** Species abundance distributions of the estimated population densities of trees in Amazon, for each data set. Total population sizes of species recorded in the sample were estimated by extrapolating distance-weighted models (see main text). Abundances are expressed in population density because the total area is different among data sets (Table SX). **Upper panel:** rank-abundance plot. **Lower panel:** octave or Preston plot (histogram of the frequency of species in abundances classes in Log2 scale).

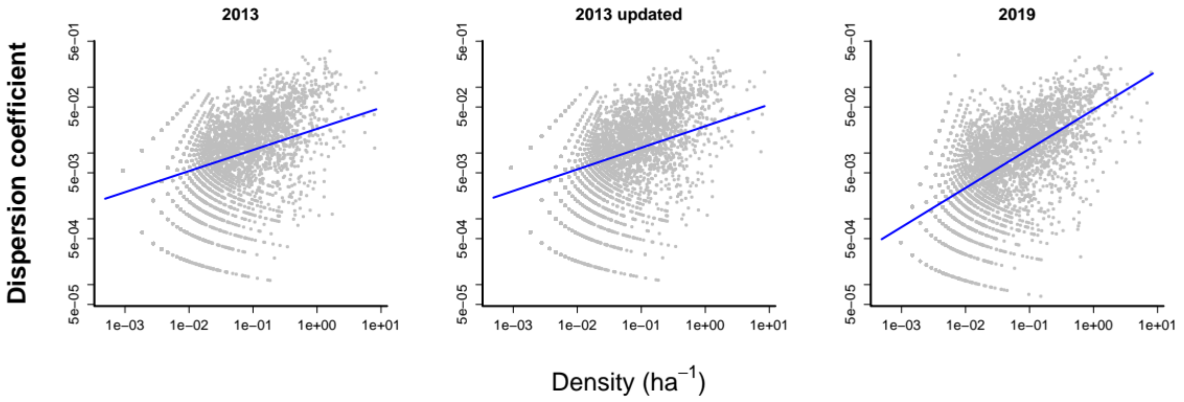


**Supplementary Fig. 8.** Estimates of spatial dispersion coefficient (k) of tree species as a function of their density, for each data set. The blue lines show the expected values of k by a linear regression in log-log scale. This regression was used to draw values of k for each species in simulated samples, with a Gaussian noise (the regression standard error) added.

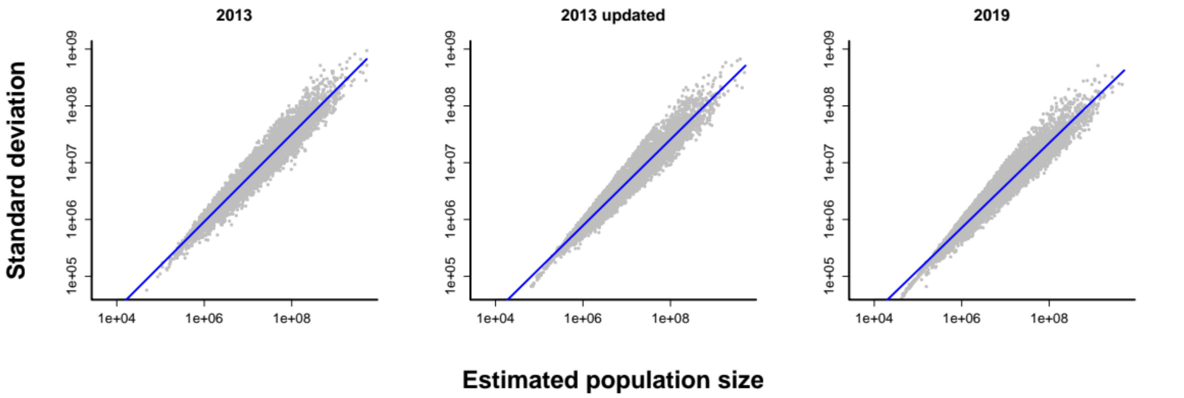


**Supplementary Fig. 9.** Regression of standard deviation of population sizes as a function of mean estimated population sizes, for each data set. The blue lines show the expected values of the standard deviation by a linear regression in log-log scale. This regression was used to draw values of SD for each species in simulated samples, with a Gaussian noise (the regression standard error) added.

# 5. Supplementary tables

**Supplementary Table 2 -** Estimates and standard errors of the parameters of SADs models fitted to the abundances in the sample of plots (TNB: Truncated negative binomial, LS: Logseries, PLN: Poisson lognormal).

| | | **2013** | | **2013 updated** | | **2019** | |
|---|---|---|---|---|---|---|---|
| | | Estimate | Std. Error | Estimate | Std. Error | Estimate | Std. Error |
| TNB | size | 0.01824 | 0.00832 | 0.04951 | 0.00887 | 0.04448 | 0.00770 |
| | mu | 12.545 | 5.356 | 32.869 | 5.014 | 52.649 | 7.824 |
| LS | alpha | 751.32 | 29.77 | 677.92 | 28.25 | 692.93 | 28.36 |
| PLN | mu | 2.2678 | 0.0555 | 2.5286 | 0.0513 | 2.8092 | 0.0504 |
| | sigma | 2.4156 | 0.0400 | 2.3271 | 0.0384 | 2.4758 | 0.0380 |

**Supplementary Table 3** - Delta-BIC for each parametric model fitted to the empirical SAD constructed using the 2013 version of the data (less plots, old taxonomy), the updated 2013 version (less plots, updated taxonomy) and the 2019 version (more plots, updated taxonomy). For each dataset version, the better supported model has 0 Delta-BIC. Models with Delta-BIC < 2 have equal support. DF= number of parameters in the model.

| **Model** | **DF** | **2013** | **2013 updated** | **2019** |
|---|---|---|---|---|
| Negative Binomial (TNB) | 2 | 4.84 | 0.00 | 0.00 |
| Logseries (LS) | 1 | 0.00 | 23.61 | 24.99 |
| Poisson-lognormal (PLN) | 2 | 65.27 | 47.87 | 42.09 |

**Supplementary Table 4 -** Point and range estimates of total number of species for each combination of data set , estimation method and bias-correction (random or clumped sampling of a LS, or none). Point estimates are maximum likelihood estimations for LS, TNB and PLN, bootstrap means for LSE, posterior means for ABC. “Lower” and “Upper” are the limits of the range estimates as follows: likelihood interval at ratio 2 for LS, TNB and PLN; 95% bootstrap empirical confidence interval for LSE; Corrected quantile of posterior distributions for ABC (see text of this supplementary material for details).

| Data set | Method | Bias correction | Point estimate | Lower | Upper |
|---|---|---|---|---|---|
| 2013 | ABC | clump | 16164 | 16145 | 16181 |
| 2013 | LS | clump | 15816 | 14659 | 17027 |
| 2013 | LS | none | 15077 | 13989 | 16215 |
| 2013 | LS | rnd | 15086 | 14018 | 16203 |
| 2013 | LSE | clump | 18056 | 17692 | 18439 |
| 2013 | LSE | none | 15196 | 14923 | 15483 |
| 2013 | LSE | rnd | 17687 | 17281 | 18113 |
| 2013 | TNB | clump | 15437 | 10461 | 19637 |
| 2013 | TNB | none | 13497 | 12448 | 14324 |
| 2013 | TNB | rnd | 16099 | 11551 | 19646 |
| 2013 updated | ABC | clump | 14941 | 14747 | 15132 |
| 2013 updated | LS | clump | 14145 | 13095 | 15244 |
| 2013 updated | LS | none | 13509 | 12486 | 14582 |
| 2013 updated | LS | rnd | 13501 | 12455 | 14598 |
| 2013 updated | LSE | clump | 15643 | 15121 | 16136 |
| 2013 updated | LSE | none | 13570 | 13339 | 13788 |
| 2013 updated | LSE | rnd | 15393 | 15094 | 15675 |
| 2013 updated | TNB | clump | 11618 | 7588 | 14881 |
| 2013 updated | TNB | none | 10271 | 9467 | 11233 |
| 2013 updated | TNB | rnd | 11725 | 9013 | 14390 |
| 2019 | ABC | clump | 15666 | 15557 | 15749 |
| 2019 | LS | clump | 14996 | 13887 | 16156 |
| 2019 | LS | none | 13793 | 12767 | 14869 |
| 2019 | LS | rnd | 13789 | 12758 | 14869 |
| 2019 | LSE | clump | 16741 | 16429 | 17020 |
| 2019 | LSE | none | 14700 | 14474 | 14902 |
| 2019 | LSE | rnd | 16527 | 16240 | 16784 |
| 2019 | TNB | clump | 13730 | 8930 | 16177 |
| 2019 | TNB | none | 10874 | 10159 | 11753 |
| 2019 | TNB | rnd | 12292 | 8910 | 15611 |

**Supplementary Table 5 - Confusion matrix of the ABC model selection for the 2013 data set, for a rejection threshold = 0.01**. The elements of the matrix show the probability of each model in the columns be selected when the data are generated by the models in the lines. The models are combinations of sampling with and without conspecific clumping (*clump* and *rnd*, respectively) of lognormal (P*LN*), logseries (*LS*) or Truncated negative binomial (*NB*). Probabilities estimated by leave-one-out cross-validation tests with 100 replicates, using the function 'cv4postpr' of the R package 'abc'.

| | LNclump | LNrnd | LSclump | LSrnd | NBclump | NBrnd |
|---|---|---|---|---|---|---|
| LNclump | 0.67 | 0.33 | 0 | 0 | 0 | 0 |
| LNrnd | 0.32 | 0.68 | 0 | 0 | 0 | 0 |
| LSclump | 0 | 0 | 0.81 | 0.18 | 0 | 0.01 |
| LSrnd | 0 | 0 | 0.19 | 0.81 | 0 | 0 |
| NBclump | 0 | 0 | 0.02 | 0 | 0.53 | 0.45 |
| NBrnd | 0 | 0 | 0.01 | 0 | 0.45 | 0.53 |

**Supplementary Table 6 -** Confusion matrix of the ABC model selection for the update 2013 data set, for a rejection threshold = 0.01. See Table S3 for details.

| | LNclump | LNrnd | LSclump | LSrnd | NBclump | NBrnd |
|---|---|---|---|---|---|---|
| LNclump | 0.68 | 0.32 | 0 | 0 | 0 | 0 |
| LNrnd | 0.26 | 0.74 | 0 | 0 | 0 | 0 |
| LSclump | 0 | 0 | 0.9 | 0.1 | 0 | 0 |
| LSrnd | 0 | 0 | 0.12 | 0.88 | 0 | 0 |
| NBclump | 0 | 0 | 0 | 0 | 0.5 | 0.49 |
| NBrnd | 0 | 0 | 0.03 | 0 | 0.47 | 0.49 |

**Supplementary Table 7 -** Confusion matrix of the ABC model selection for the update 2013 data set, for a rejection threshold = 0.01. See Table S3 for details.

| | LNclump | LNrnd | LSclump | LSrnd | NBclump | NBrnd |
|---|---|---|---|---|---|---|
| LNclump | 0.92 | 0.08 | 0 | 0 | 0 | 0 |
| LNrnd | 0.11 | 0.89 | 0 | 0 | 0 | 0 |
| LSclump | 0 | 0 | 0.95 | 0.04 | 0 | 0.01 |
| LSrnd | 0 | 0 | 0.05 | 0.95 | 0 | 0 |
| NBclump | 0 | 0 | 0.02 | 0 | 0.52 | 0.46 |
| NBrnd | 0 | 0 | 0.03 | 0 | 0.45 | 0.52 |

## 6. ATDN 2019 data set (Supplementary Fig. 10, 11)

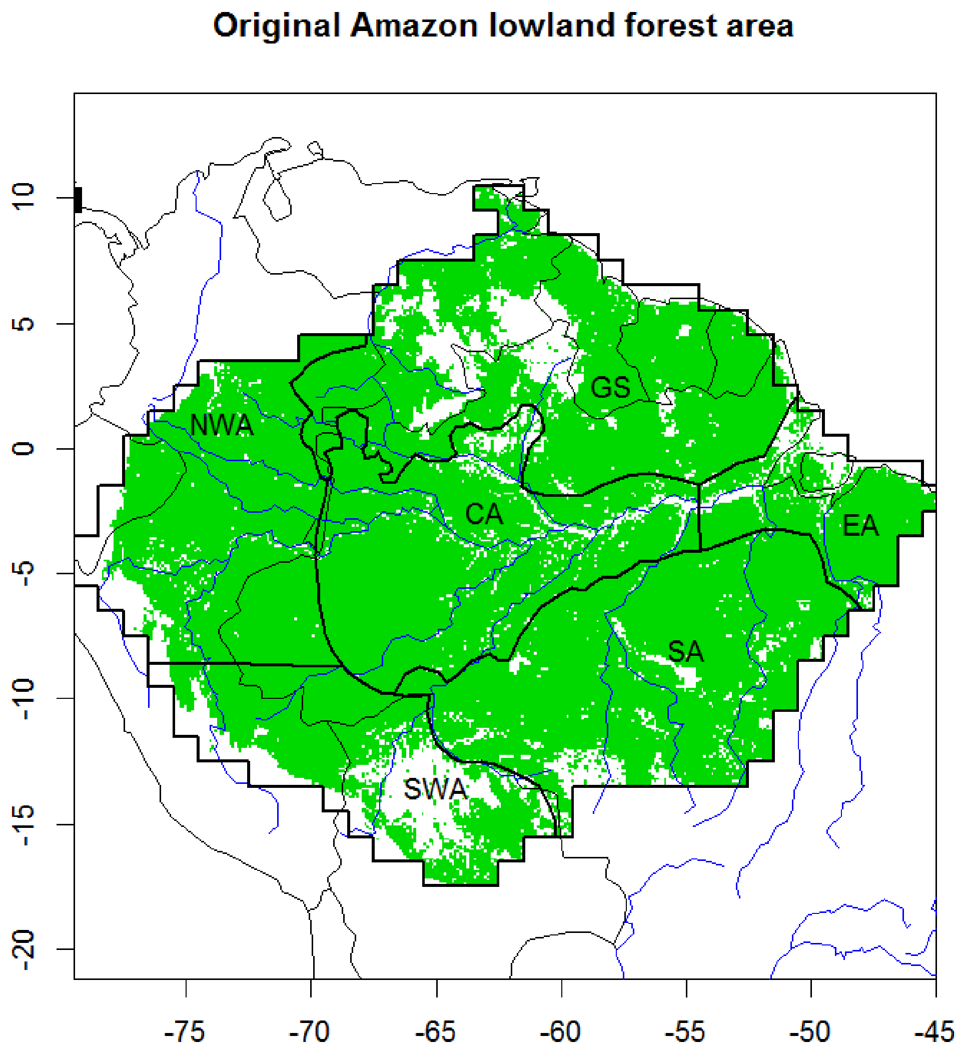


**Supplementary Fig. 10**. **Map of lowland forest in the Amazon**. The green area represents land area [22] minus large water bodies [22], areas over 500 m elevation [23], and areas originally without forest [24,25] and is approximately 5.79 million km$^2$. Regions: CA, central Amazonia; EA, eastern Amazonia; GS, Guiana Shield; SA, southern Amazonia; NWA, northwestern Amazonia; SWA, southwestern Amazonia. Map created with custom R [26] script. Base map source (country.shp, rivers.shp): ESRI (http://www.esri.com/data/basemaps, © Esri, DeLorme Publishing Company).

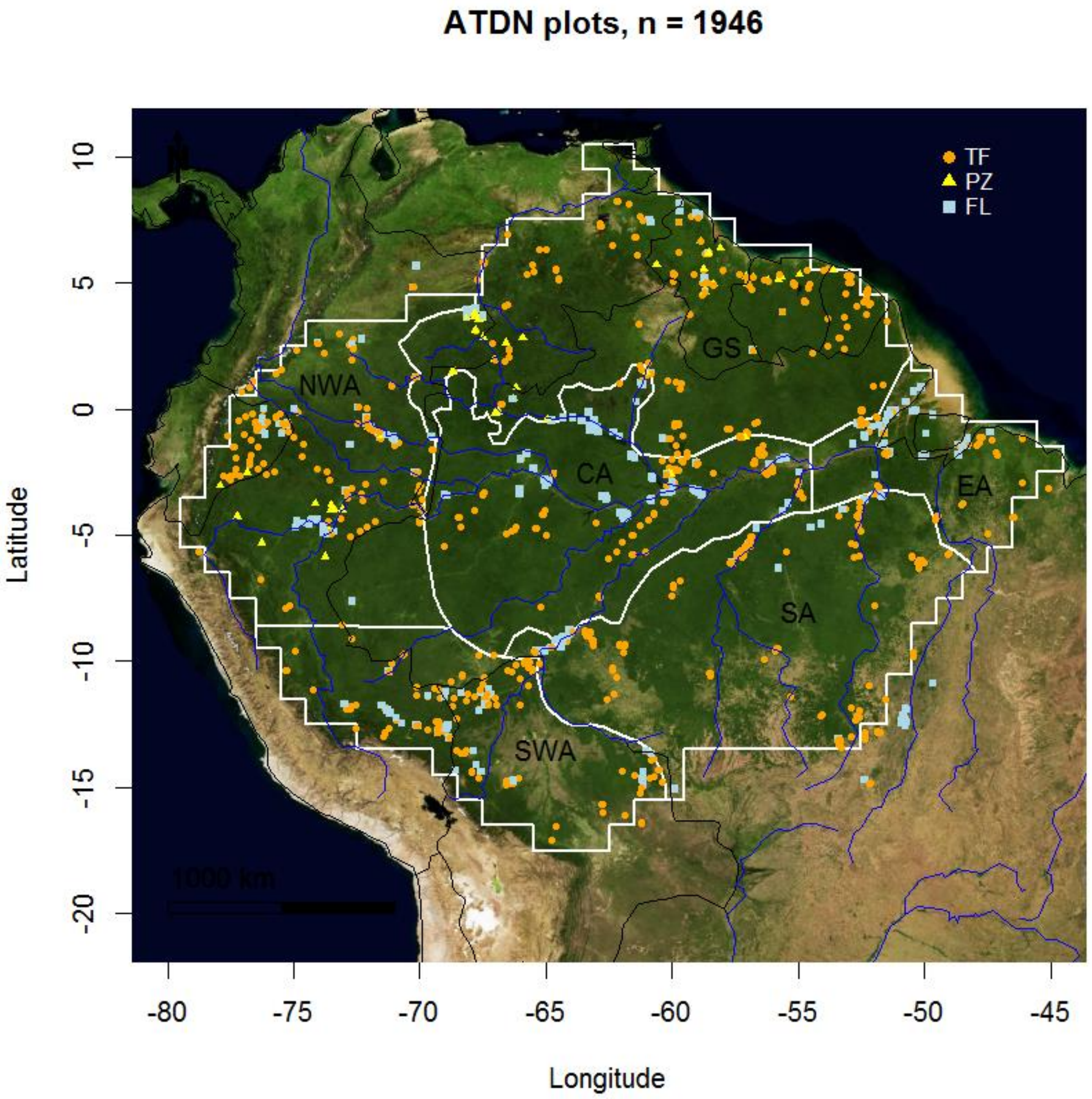


**Supplementary Fig. 11. A map of Amazonia showing the location of the 1,946 Amazon Tree Diversity Network (ATDN) plots that contributed data to this paper**. The white polygon marks our delimitation of the study area. Orange circles indicate plots on terra firme; blue squares, plots on seasonally or permanently flooded terrain (várzea, igapó, swamps); yellow triangles, plots on white-sand podzols; gray circles, plots only used for tree density calculations. Plots outside the Amazon polygon were used to improve interpolation of populations near the edges. Background is from Visible Earth. Regions: CA, central Amazonia; EA, eastern Amazonia; GS, Guiana Shield; SA, southern Amazonia; NWA, northwestern Amazonia; SWA, southwestern Amazonia. Map created with custom R[26] script. Base map source (country.shp, rivers.shp): ESRI (http://www.esri.com/data/basemaps, © Esri, DeLorme Publishing Company). Background: Visible Earth NASA (https://visibleearth.nasa.gov/useterms.php © NASA).

## 7. Tree densities (Supplementary Fig. 12)

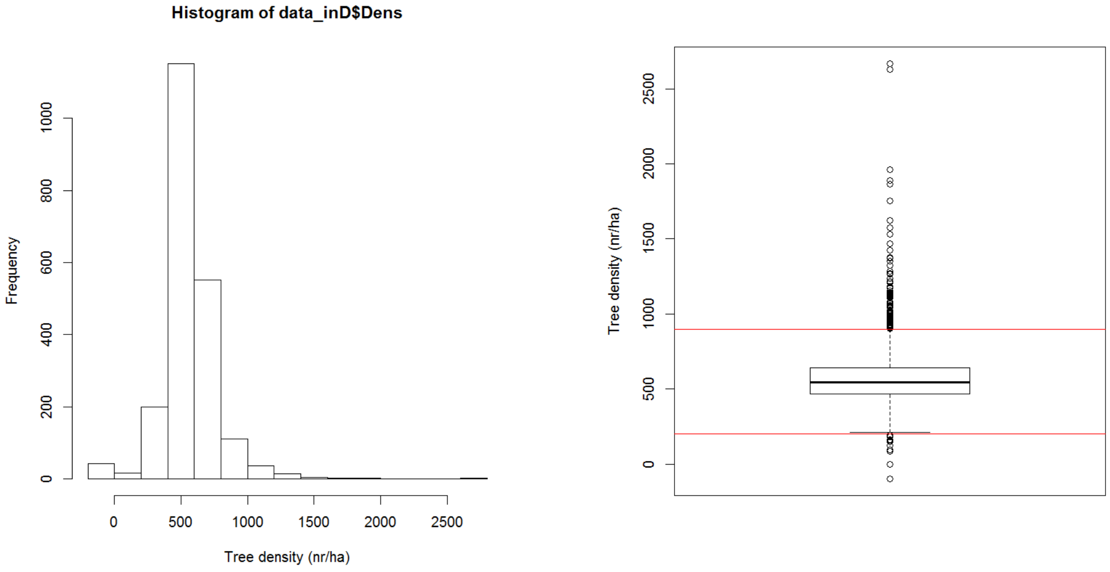


**Supplementary Fig. 12. Histogram and boxplot of tree density per ha for the 2220 forest plots with known tree density across the Amazon forest, for the 2019 data set**. In the boxplot on the right panel, the cut-off levels of 200 and 900 trees per ha are given as red lines, which are approximately equivalent to the outlier lines of the boxplot. Fifty-nine and 105 plots had tree densities below and above the outlier levels respectively.

# 8. References


1 ter Steege, H. *et al.* Hyperdominance in the Amazonian tree flora. *Science* **342**, 1243092, doi:10.1126/science.1243092 (2013).

2 Ulrich, W. & Ollik, M. Limits to the estimation of species richness: the use of relative abundance distributions. *Diversity and Distributions* **11**, 265-273 (2005).

3 Baldridge, E., Harris, D. J., Xiao, X. & White, E. P. An extensive comparison of species-abundance distribution models. *PeerJ* **4**, e2823, doi:10.7717/peerj.2823 (2016).

4 ter Steege, H. *et al.* Estimating the global conservation status of over 15,000 Amazonian tree species. *Science Advances* **1**, e1500936, doi:10.1126/sciadv.1500936 (2015).

5 Fisher, R. A., Corbet, A. S. & Williams, C. B. The relation between the number of species and the number of individuals in a random sample of an animal population. *Journal of Animal Ecology* **12**, 42-58 (1943).

6 Alonso, D., Ostling, A. & Etienne, R. S. The implicit assumption of symmetry and the species abundance distribution. *Ecology Letters* **11**, 93-105 (2008).

7 Green, J. L. & Plotkin, J. B. A statistical theory for sampling species abundances. *Ecology Letters* **10**, 1037-1045 (2007).

8 Pielou, E. C. *Mathematical Ecology*. (John Wiley and Sons, 1977).

9 Preston, F. W. The commonness, and rarity of species. *Ecology* **29**, 254-283, doi:doi:10.2307/1930989 (1948).

10 Saether, B. E., Engen, S. & Grøtan, V. Species diversity and community similarity in fluctuating environments: parametric approaches using species abundance distributions. *The Journal of Animal Ecology.* **82**, 721-738 (2013).

11 Bulmer, M. G. On fitting the Poisson lognormal distribution to species abundance data. *Biometrics* **30**, 101-110 (1974).

12 Tovo, A. *et al.* Upscaling species richness and abundances in tropical forests. *Science Advances* **3**, e1701438, doi:10.1126/sciadv.1701438 (2017).

13 Prado, P. I., Miranda, M. D. & Chalom, A. sads: Maximum likelihood models for species abundance distributions. (CRAN network, https://CRAN.R-project.org/package=sads, 2018).

14 Chao, A. Estimating population size for sparse data in capture-recapture experiments. *Biometrics* **45**, 427-438 (1989).

15 Burnham, K. P. & Anderson, D. R. *Model selection and multimodel inference - A practical-theoretic approach*. (Springer-Verlag, 2002).

16 Edwards, A. W. F. *Likelihood: An Account of the Statistical Concept of Likelihood and its Application to Scientific Inference*. (Cambridge University Press, 1972).

17 Condit, R. *et al.* Spatial patterns in the distribution of tropical tree species. *Science* **288**, 1414, doi:10.1126/science.288.5470.1414 (2000).

18 Plotkin, J. B. & Muller-Landau, H. C. Sampling the species composition of a landscape. *Ecology* **83**, 3344-3356, doi:10.1890/0012-9658 (2002).

19 Plotkin, J. B. *et al.* Species-area curves, spatial aggregation, and habitat specialization in tropical forests. *Journal of Theoretical Biology* **207**, 81-99, doi:https://doi.org/10.1006/jtbi.2000.2158 (2000).

20 Csilléry, K., Blum, M. G. B., Gaggiotti, O. E. & François, O. Approximate Bayesian Computation (ABC) in practice. *Trends in Ecology & Evolution* **25**, 410-418, doi:https://doi.org/10.1016/j.tree.2010.04.001 (2010).

21 Csilléry, K., François, O. & Blum, M. G. B. abc: an R package for approximate Bayesian computation (ABC). *Methods in Ecology and Evolution* **3**, 475-479, doi:10.1111/j.2041-210X.2011.00179.x (2012).

22 Environmental Systems Research Institute. ESRI Data & Maps 1999 - An ESRI White Paper. (Environmental Systems Research Institute, Redlands, US, 1999).

23 Shuttle Radar Propulsion Mission. NASA Jet Propulsion Laboratory. <http://www2.jpl.nasa.gov/srtm/> (2009).

24 Soares-Filho, B. S. *et al.* Modelling conservation in the Amazon basin. *Nature* **440**, 520-523, doi:http://www.nature.com/nature/journal/v440/n7083/suppinfo/nature04389_S1.html (2006).

25 Soares-Filho, B. S. *et al.* (http://daac.ornl.gov//LBA/guides/LC14_Amazon_Scenarios.html, 2013).

26 R Development Core Team. R: A language and environment for statistical computing. Report No. ISBN 3-900051-07-0, (R Foundation for Statistical Computing, Vienna, Austria, 2017).

**Affiliations**

1Naturalis Biodiversity Center, PO Box 9517, Leiden, 2300 RA, The Netherlands
2Systems Ecology, Vrije Universiteit Amsterdam, De Boelelaan 1087, Amsterdam, 1081 HV, The Netherlands
3Instituto de Biociências - Dept. Ecologia, Universidade de Sao Paulo - USP, Rua do Matão, Trav. 14, no. 321, Cidade Universitária, São Paulo, SP, 05508-090, Brazil
4Ecology & Biodiversity Group, Utrecht University, Padualaan 8, Utrecht, 3584 CH, The Netherlands
5Coordenação de Biodiversidade, Instituto Nacional de Pesquisas da Amazônia - INPA, Av. André Araújo, 2936, Petrópolis, Manaus, AM, 69067-375, Brazil
6Programa Professor Visitante Nacional Sênior na Amazônia - CAPES, Universidade Federal Rural da Amazônia, Av. Perimetral, s/n, Belém, PA, Brazil
7Coordenação de Botânica, Museu Paraense Emílio Goeldi, Av. Magalhães Barata 376, C.P. 399, Belém, PA, 66040-170, Brazil
8EMBRAPA – Centro de Pesquisa Agroflorestal de Roraima, BR 174, km 8 – Distrito Industrial, Boa Vista, RR, 69301-970, Brazil
9School of Geography, University of Leeds, Woodhouse Lane, Leeds, LS2 9JT, UK
10Grupo de Investigación en Biodiversidad, Medio Ambiente y Salud-BIOMAS, Universidad de las Américas, Campus Queri, Quito, Ecuador
11Keller Science Action Center, The Field Museum, 1400 S. Lake Shore Drive, Chicago, IL, 60605-2496, USA
12Departamento de Botânica, Instituto de Pesquisas Científicas e Tecnológicas do Amapá - IEPA, Rodovia JK, Km 10, Campus do IEPA da Fazendinha, Amapá, 68901-025, Brazil
13Herbario Amazónico Colombiano, Instituto SINCHI, Calle 20 No 5-44, Bogotá, DC, Colombia
14Coordenação de Pesquisas em Ecologia, Instituto Nacional de Pesquisas da Amazônia - INPA, Av. André Araújo, 2936, Petrópolis, Manaus, AM, 69067-375, Brazil
15Dep. of Wetland Ecology, Institute of Geography and Geoecology, Karlsruhe Institute of Technology - KIT, Josefstr.1, Rastatt, D-76437, Germany
16Biogeochemistry, Max Planck Institute for Chemistry, Hahn-Meitner Weg 1, Mainz, 55128, Germany
17AMAP, IRD, Cirad, CNRS, INRA, Université de Montpellier, Montpellier, F-34398, France
18Coordenação de Dinâmica Ambiental, Instituto Nacional de Pesquisas da Amazônia - INPA, Av. André Araújo, 2936, Petrópolis, Manaus, AM, 69067-375, Brazil
19Science and Education, The Field Museum, 1400 S. Lake Shore Drive, Chicago, IL, 60605-2496, USA
20Jardín Botánico de Missouri, Oxapampa, Pasco, Peru
21Centro de Biociências, Departamento de Ecologia, Universidade Federal do Rio Grande do Norte, Av. Senador Salgado Filho, 3000 , Natal, RN, 59072-970, Brazil
22Departamento de Ecologia, Universidade Estadual Paulista - UNESP – Instituto de Biociências – IB, Av. 24 A, 1515, Bela Vista, Rio Claro, SP, 13506-900, Brazil
23Herbario Vargas, Universidad Nacional de San Antonio Abad del Cusco, Avenida de la Cultura, Nro 733, Cusco, Cuzco, Peru
24Biological and Environmental Sciences, University of Stirling, Stirling, FK9 4LA, UK
25Divisao de Sensoriamento Remoto – DSR, Instituto Nacional de Pesquisas Espaciais – INPE, Av. dos Astronautas, 1758, Jardim da Granja, São José dos Campos, SP, 12227-010, Brazil
26Programa de Pós- Graduação em Biodiversidade e Biotecnologia PPG- Bionorte, Universidade Federal de Rondônia, Campus Porto Velho Km 9,5 bairro Rural, Porto Velho, RO, 76.824-027, Brazil
27Department of Biology and Florida Museum of Natural History, University of Florida, Gainesville, FL, 32611, USA
28Centre for Tropical Environmental and Sustainability Science and College of Science and Engineering, James Cook University, Cairns, Queensland, 4870, Australia

29Departamento de Biologia, Universidade Federal de Rondônia, Rodovia BR 364 s/n Km 9,5 - Sentido Acre, Unir, Porto Velho, RO, 76.824-027, Brazil
30Instituto de Investigaciones de la Amazonía Peruana (IIAP), Av. A. Quiñones km 2,5, Iquitos, Loreto, 784, Peru
31Instituto Boliviano de Investigacion Forestal, Av. 6 de agosto #28, Km. 14, Doble via La Guardia, Casilla 6204, Santa Cruz, Santa Cruz, Bolivia
32Departamento de Ciencias Forestales, Universidad Nacional de Colombia, Calle 64 x Cra 65, Medellín, Antioquia, 1027, Colombia
33Agteca-Amazonica, Santa Cruz, Bolivia
34Programa de Pós-Graduação em Ecologia e Conservação, Universidade do Estado de Mato Grosso, Nova Xavantina, MT, Brazil
35Facultad de Ciencias Agrícolas, Universidad Autónoma Gabriel René Moreno, Santa Cruz, Santa Cruz, Bolivia
36Geography, College of Life and Environmental Sciences, University of Exeter, Rennes Drive, Exeter, EX4 4RJ, UK
37International Center for Tropical Botany (ICTB) Department of Biological Sciences, Florida International University, 11200 SW 8th Street, OE 243, Miami, FL, 33199, USA
38Cirad UMR Ecofog, AgrosParisTech,CNRS,INRA,Univ Guyane, Campus agronomique, Kourou Cedex, 97379, France
39Natural History Museum, University of Oslo, Postboks 1172, Oslo, 0318, Norway
40Centro de Investigaciones Ecológicas de Guayana, Universidad Nacional Experimental de Guayana, Calle Chile, urbaniz Chilemex, Puerto Ordaz, Bolivar, Venezuela
41Prédio da Botânica e Ecologia, Embrapa Recursos Genéticos e Biotecnologia, Parque Estação Biológica, Av. W5 Norte, Brasilia, DF, 70770-917, Brazil
42Projeto Dinâmica Biológica de Fragmentos Florestais, Instituto Nacional de Pesquisas da Amazônia - INPA, Av. André Araújo, 2936, Petrópolis, Manaus, AM, 69067-375, Brazil
43Diretoria Técnico-Científica, Instituto de Desenvolvimento Sustentável Mamirauá, Estrada do Bexiga, 2584, Tefé, AM, 69470-000, Brazil
44Instituto de Ciências Biológicas, Universidade Federal do Pará, Av. Augusto Corrêa 01, Belém, PA, 66075-110, Brazil
45Laboratório de Ecologia de Doenças Transmissíveis da Amazônia (EDTA), Instituto Leônidas e Maria Deane, Fiocruz, Rua Terezina, 476, Adrianópolis, Manaus, AM, 69060-001, Brazil
46Programa de Pós-graduação em Biodiversidade e Saúde, Instituto Oswaldo Cruz - IOC/FIOCRUZ, Pav. Arthur Neiva – Térreo, Av. Brasil, 4365 – Manguinhos, Rio de Janeiro, RJ, 21040-360, Brazil
47Programa de Pós-Graduação em Ecologia, Universidade Federal do Pará, Av. Augusto Corrêa 01, Belém, PA, 66075-110, Brazil
48Embrapa Amazônia Oriental, Trav. Dr. Enéas Pinheiro s/nº, Belém, PA, 66095-100, Brazil
49Programa de Ciencias del Agro y el Mar, Herbario Universitario (PORT), UNELLEZ-Guanare, Guanare, Portuguesa, 3350, Venezuela
50Instituto de Biociências - Dept. Botanica, Universidade de Sao Paulo - USP,  Rua do Matão 277, Cidade Universitária, São Paulo, SP, 05508-090, Brazil
51Laboratorio de Ecología de Bosques Tropicales y Primatología, Universidad de los Andes, Carrera 1 # 18a- 10, Bogotá, DC, 111711, Colombia
52Programa de Pós-Graduação em Biologia (Botânica), Instituto Nacional de Pesquisas da Amazônia - INPA, Av. André Araújo, 2936, Petrópolis, Manaus, AM, 69067-375, Brazil
53Institute of Biodiversity and Ecosystem Dynamics, University of Amsterdam, Sciencepark 904, Amsterdam, 1098 XH, The Netherlands
54Endangered Species Coalition, 8530 Geren Rd., Silver Spring, MD, 20901, USA
55Museo de Historia Natural Noel Kempff Mercado, Universidad Autónoma Gabriel Rene Moreno, Avenida Irala 565 Casilla Post al 2489, Santa Cruz, Santa Cruz, Bolivia
56Facultad de Ciencias Forestales y Ambientales, Instituto de Investigaciones para el Desarrollo

Forestal, Universidad de los Andes, Via Chorros de Milla, 5101, Mérida, Mérida, Venezuela
57Inventory and Monitoring Program, National Park Service, 120 Chatham Lane, Fredericksburg, VA, 22405, USA
58Center for Conservation and Sustainability, Smithsonian Conservation Biology Institute, 1100 Jefferson Dr. SW, Suite 3123, Washington, DC, 20560-0705, USA
59Universidade Federal do Amapá, Ciências Ambientais, Rod. Juscelino Kubitschek km2, Macapá, AP, 68902-280, Brazil
60Department of Integrative Biology, University of California, Berkeley, CA, 94720-3140, USA
61Biologia Vegetal, Universidade Estadual de Campinas, Caixa Postal 6109, Campinas, SP, 13.083-970, Brazil
62Department of Ecology, University of Brasilia, Brasilia, DF, 70904-970, Brazil
63Department of Ecology and Evolutionary Biology, Cornell University, Corson Hall, 215 Tower Road, Ithaca, NY, 14850, USA
64Peruvian Center for Biodiversity and Conservation (PCBC), Iquitos, Peru
65Department of Global Ecology, Carnegie Institution for Science, 260 Panama St., Stanford, CA, 94305, USA
66Andes to Amazon Biodiversity Program, Madre de Dios, Madre de Dios, Peru
67Faculty of Natural Sciences, Department of Life Sciences, Imperial College London, Silwood Park, South Kensington Campus, London, SW7 2AZ, UK
68Escola de Negócios Tecnologia e Inovação, Centro Universitário do Pará, Belém, PA, Brazil
69Universidade Federal do Pará, Rua Augusto Corrêa 01, Belém, PA, 66075-110, Brazil
70Ecosistemas, Biodiversidad y Conservación de Especies, Universidad Estatal Amazónica, Km. 2 1/2 vía a Tena (Paso Lateral), Puyo, Pastaza, Ecuador
71Universidade Federal de Minas Gerais, Instituto de Ciências Biológicas, Departamento de Genética, Ecologia e Evolução, Av. Antônio Carlos, 6627 Pampulha, Belo Horizonte, MG, 31270-901, Brazil
72Department of Biology, University of Miami, Coral Gables, FL, 33146, USA
73Fairchild Tropical Botanic Garden, Coral Gables, FL, 33156, USA
74Environmental Change Institute, University of Oxford, Oxford, Oxfordshire, OX1 3QY, UK
75Lancaster Environment Centre, Lancaster University, Lancaster, Lancashire, LA1 4YQ, UK
76Empresa Brasileira de Pesquisa Agropecuária, Embrapa Amapá, Rod. Juscelino Kubitschek km 5, Macapá, Amapá, 68903-419, Brazil
77Direccíon de Evaluación Forestal y de Fauna Silvestre, Av. Javier Praod Oeste 693, Magdalena del Mar, Peru
78Universidad Regional Amazónica IKIAM, Km 7 via Muyuna, Tena, Napo, Ecuador
79Grupo de Ecología y Conservación de Fauna y Flora Silvestre, Instituto Amazónico de Investigaciones Imani, Universidad Nacional de Colombia sede Amazonia, Leticia, Amazonas, Colombia
80Escuela de Biología Herbario Alfredo Paredes, Universidad Central, Ap. Postal 17.01.2177, Quito, Pichincha, Ecuador
81Iwokrama International Centre for Rain Forest Conservation and Development, Georgetown, Guyana
82Museu Universitário / Centro de Ciências Biológicas e da Natureza / Laboratório de Botânica e Ecologia Vegetal, Universidade Federal do Acre, Rio Branco, AC, 69915-559, Brazil
83Department of Biological Sciences, Humboldt State University, 1 Harpst Street, Arcata, CA, 95521, USA
84Institute of Biological and Health Sciences, Federal University of Alagoas, Av. Lourival Melo Mota, s/n, Tabuleiro do Martins, Maceio, AL, 57072-970, Brazil
85Servicios de Biodiversidad EIRL, Jr. Independencia 405, Iquitos, Loreto, 784, Peru
86School of Geosciences, University of Edinburgh, 201 Crew Building, King's Buildings, Edinburgh, EH9 3JN, UK

87Tropical Diversity Section, Royal Botanic Garden Edinburgh, 20a Inverleith Row, Edinburgh, Scotland, EH3 5LR, UK
88New York Botanical Garden, 2900 Southern Blvd, Bronx, New York, NY, 10458-5126, USA
89Natural Capital and Plant Health, Royal Botanic Gardens, Kew, Richmond, Surrey, TW9 3AB, UK
90Center for Conservation and Sustainable Development, Missouri Botanical Garden, P.O. Box 299, St. Louis, MO, 63166-0299, USA
91Universidad Nacional de Jaén, Carretera Jaén San Ignacio Km 23, Jaén, Cajamarca, 06801, Peru
92School of Environmental Sciences, University of East Anglia, Norwich, NR4 7TJ, UK
93Department for Identification & Naming, Royal Botanic Gardens, Kew, Richmond, Surrey, TW9 3AB, UK
94Herbario Nacional de Bolivia, Universitario UMSA, Casilla 10077 Correo Central, La Paz, La Paz, Bolivia
95Biology Department and Center for Energy, Environment and Sustainability, Wake Forest University, 1834 Wake Forest Rd, Winston Salem, NC, 27106, USA
96Department of Anthropology, University of Texas at Austin, SAC 5.150, 2201 Speedway Stop C3200, Austin, TX, 78712, USA
97Fundación Estación de Biología, Cra 10 No. 24-76 Oficina 1201, Bogotá, DC, Colombia
98Laboratoire Evolution et Diversité Biologique, CNRS and Université Paul Sabatier, UMR 5174 EDB, Toulouse, 31000, France
99Biosystematics group, Wageningen University, Droevendaalsesteeg 1, Wageningen, 6708 PB, The Netherlands
100Fundación Puerto Rastrojo, Cra 10 No. 24-76 Oficina 1201, Bogotá, DC, Colombia
101Colegio de Ciencias Biológicas y Ambientales-COCIBA & Galapagos Institute for the Arts and Sciences-GAIAS, Universidad San Francisco de Quito-USFQ, Quito, Pichincha, Ecuador
102Department of Wildlife Ecology and Conservation, University of Florida, 110 Newins-Ziegler Hall, Gainesville, FL, 32611, USA
103ICNHS, Federal University of Mato Grosso, Av. Alexandre Ferronato 1200, Setor Industrial, Sinop, MT, 78.557-267, Brazil
104Instituto de Ciencias Naturales, Universidad Nacional de Colombia, Apartado 7945, Bogotá, DC, Colombia
105ACEER Foundation, Jirón Cusco N° 370, Puerto Maldonado, Madre de Dios, Peru
106PROTERRA, Instituto de Investigaciones de la Amazonía Peruana (IIAP), Av. A. Quiñones km 2,5, Iquitos, Loreto, 784, Peru
107Laboratory of Human Ecology, Instituto Venezolano de Investigaciones Científicas - IVIC, Ado 20632, Caracas, DC, 1020A, Venezuela
108Universidad Autónoma del Beni José Ballivián, Campus Universitario Final, Av. Ejercito, Riberalta, Beni, Bolivia
109Direction régionale de la Guyane, ONF, Cayenne, F-97300, French Guiana
110Institut de Ciència i Tecnologia Ambientals, Universitat Autònoma de Barcelona, 08193 Bellaterra, Barcelona, Spain
111Amazon Conservation Team, Doekhieweg Oost #24, Paramaribo, Suriname
112Environmental Change Institute, Oxford University Centre for the Environment, Dyson Perrins Building, South Parks Road, Oxford, England, OX1 3QY, UK
113Instituto de Ciência Agrárias, Universidade Federal Rural da Amazônia, Av. Presidente Tancredo Neves 2501, Belém, PA, 66.077-830, Brazil
114Escuela Profesional de Ingeniería Forestal, Universidad Nacional de San Antonio Abad del Cusco, Jirón San Martín 451, Puerto Maldonado, Madre de Dios, Peru
115Department of Ecology and Evolutionary Biology, University of Michigan, Ann Arbor, MI, 48109, USA
116University of Nottingham, University Park, Nottingham, NG7 2RD, UK
117Geography and the Environment, University of Texas at Austin, 305 E. 23rd Street, CLA building,

Austin, TX, 78712, USA
118GeoIS, El Día 369 y El Telégrafo, 3° Piso, Quito, Pichincha, Ecuador
119School of Agriculture and Food Sciences - ARC Centre of Excellence for Environmental Decisions CEED, The University of Queensland, St. Lucia, QLD 4072, Australia
120Instituto de Investigaciones para el Desarrollo Forestal (INDEFOR), Universidad de los Andes, Conjunto Forestal, 5101, Mérida, Mérida, Venezuela
121School of Environmental and Forest Sciences, University of Washington, Seattle, WA, 98195-2100, USA
122Environmental Science and Policy, Northern Arizona University, Flagstaff, AZ, 86011, USA
123Laurentian University, 935 Ramsey Lake Road, Sudbury, Ontario, P3E 2C6, Canada
124Resource Ecology Group, Wageningen University & Research, Droevendaalsesteeg 3a, Lumen, building number 100, Wageningen, Gelderland, 6708 PB, The Netherlands
125Laboratório de Ciências Ambientais, Universidade Estadual do Norte Fluminense, Av. Alberto Lamego 2000, Campos dos Goyatacazes, RJ, 28013-620, Brazil
126University of Campinas, Plant Biology Department, Rua Monteiro Lobato, 255, Cidade Universitária Zeferino Vaz, Barão Geraldo, Campinas, São Paulo, CEP 13083-862, Brazil
127Departamento de Biologia, Universidade Federal do Amazonas - UFAM – Instituto de Ciências Biológicas – ICB1, Av General Rodrigo Octavio 6200, Manaus, AM, 69080-900, Brazil
128Cambridge University Botanic Garden, 1 Brookside., Cambridge, CB2 1JE, UK
129Medio Ambiente, PLUSPRETOL, Iquitos, Loreto, Peru
130The Mauritius Herbarium, Agricultural Services, Ministry of Agro-Industry and Food Security, Reduit, 80835, Mauritius
131Escuela de Ciencias Forestales (ESFOR), Universidad Mayor de San Simon (UMSS), Sacta, Cochabamba, Bolivia
132FOMABO, Manejo Forestal en las Tierras Tropicales de Bolivia, Sacta, Cochabamba, Bolivia
133Tropenbos International, Lawickse Allee 11 PO Box 232, Wageningen, 6700 AE, The Netherlands
134Living Earth Collaborative, Washington University in Saint Louis, St. Louis, MO, 63130, USA
135Department of Bioscience, Aarhus University, Building 1540 Ny Munkegade, Aarhus C, Aarhus, DK-8000, Denmark
136School of Anthropology and Conservation, University of Kent, Marlowe Building, Canterbury, Kent, CT2 7NR, UK
137Department of Biology, University of Missouri, St. Louis, MO, 63121, USA
138Facultad de Biologia, Universidad Nacional de la Amazonia Peruana, Pevas 5ta cdra, Iquitos, Loreto, Peru
139Herbario Nacional del Ecuador, Universidad Técnica del Norte, Quito, Pichincha, Ecuador
140Instituto de Biodiversidade e Floresta, Universidade Federal do Oeste do Pará, Rua Vera Paz, Campus Tapajós, Santarém, PA, 68015-110, Brazil
